# Benchmarking overlapping community detection methods for applications in human connectomics

**DOI:** 10.1162/NETN.a.39

**Published:** 2026-01-08

**Authors:** Annie G. Bryant, Aditi Jha, Sumeet Agarwal, Patrick Cahill, Brandon Lam, Stuart Oldham, Aurina Arnatkevičiūtė, Alex Fornito, Ben D. Fulcher

**Affiliations:** School of Physics, The University of Sydney, Camperdown, NSW, Australia; Centre for Complex Systems, The University of Sydney, Camperdown, NSW, Australia; Department of Statistics and the Wu Tsai Neuroscience Institute, Stanford University, Stanford, CA, USA; Yardi School of Artificial Intelligence, Indian Institute of Technology Delhi, New Delhi, India; School of Psychological Sciences, Turner Institute for Brain and Mental Health & Monash Biomedical Imaging, Monash University, Clayton, VIC, Australia; Developmental Imaging, Murdoch Children’s Research Institute, Parkville, VIC, Australia

**Keywords:** Brain networks, Community detection, Structural connectome, Diffusion MRI

## Abstract

Brain networks exhibit non-trivial modular organization, with groups of densely connected areas participating in specialized functions. Traditional community detection algorithms assign each node to one module, but this representation cannot capture integrative, multifunctional nodes that span multiple communities. Despite the increasing availability of overlapping community detection algorithms (OCDAs) to capture such integrative nodes, there is no objective procedure for selecting the most appropriate method and its parameters for a given problem. Here, we overcome this limitation by introducing a data-driven method for selecting an OCDA and its parameters from performance on a tailored ensemble of generated benchmark networks, assessing 22 unique algorithms and parameter settings. Applied to the human right-hemisphere structural connectome, we find that the “order statistics local optimization method” (OSLOM) best identifies ground-truth overlapping structure in the benchmark ensemble, yielding a seven-network decomposition of the right-hemisphere cortex. These modules are bridged by 15 overlapping regions that generally sit at the apex of the putative cortical hierarchy—suggesting integrative, higher order function—with network participation increasing along the cortical hierarchy, a finding not supported using a non-overlapping modular decomposition. This data-driven approach to selecting OCDAs is applicable across domains, opening new avenues to detecting and quantifying informative structures in complex real-world networks.

## Author Summary

The cortical connectome is characterized by densely interconnected modules or “communities” of brain regions. While traditional algorithms for partitioning regions into modules force each region into a single module, overlapping community detection algorithms (OCDAs) can capture more complex structures in which regions can overlap among multiple communities. Here, we introduce a new data-driven approach to identify the most suitable OCDA for a given network, which we apply to the cortical connectome. Our overlapping partition reveals important aspects of network architecture that are missing in non-overlapping representations, with overlapping regions (with multiple community assignments) mostly situated high in the cortical hierarchy, consistent with their integrative role. Our results provide new insights into the multifaceted organization of the human connectome, and our method could be used to uncover new types of structure in diverse complex systems in the future.

## INTRODUCTION

The complex web of structural connections between brain areas can be represented as a network of nodes (brain regions) and edges (axonal connections; Bullmore & Sporns, 2012; Fornito, Zalesky, & Bullmore, 2016). This representation allows us to quantify and understand properties of the brain’s network structure, like its organization into densely interconnected modules. Connectome modules often correspond to segregated groups of brain regions with distinct, functionally specialized roles (Betzel et al., 2015; Sporns & Betzel, 2016), wherein highly connected hub nodes are thought to play an integrative role in communicating diverse information between them (van den Heuvel, Kahn, Goñi, & Sporns, 2012). Accurately delineating network modules is an essential step in understanding the brain’s modular organization. The community detection algorithms that have most commonly been used to study brain network modules have assumed a hard partition of nodes into mutually exclusive communities, that is, each node is assigned to a single community. This non-overlapping modular decomposition can be represented as a discrete categorical label that captures each node’s modular affiliation (Fortunato, 2010).

Applications of community detection to brain networks have ranged from the structural network of individual neurons in *C. elegans* (Towlson, Vértes, Ahnert, Schafer, & Bullmore, 2013) and *Drosophila* (Shih et al., 2015) to the mesoscopic axonal connections in mouse (Rubinov, Ypma, Watson, & Bullmore, 2015; Q. Wang, Sporns, & Burkhalter, 2012; Zingg et al., 2014), non-human primate (Harriger, van den Heuvel, & Sporns, 2012), and human (Bertolero, Yeo, & D’Esposito, 2015; Betzel, Fukushima, He, Zuo, & Sporns, 2016; Betzel et al., 2017) brains, where inferred network modules align with broad specializations of cognitive function (Crossley et al., 2013). While these studies support a conceptual model of the brain in which segregated groups of nodes process specialized types of information, non-overlapping partitions present limitations that may yield unintended consequences for inferring network-wide modular organization—for example, collapsing two modules bridged by one or more overlapping nodes into a single module. Indeed, transmodal cortical association areas are defined by their flexible multifaceted functions and might, therefore, be more accurately affiliated with multiple modules. A representation that allows nodes to belong to multiple communities may thus provide a more complete picture of the brain’s structural and functional organization (Najafi, McMenamin, Simon, & Pessoa, 2016; Vafaii et al., 2024).

Overlapping community detection algorithms (OCDAs) decompose the network into a fixed number of modules, as non-overlapping methods do, but with the additional freedom of allowing each node to belong to multiple communities. A wide range of such methods have been developed (Lancichinetti, Radicchi, Ramasco, & Fortunato, 2011; Liu, Zhang, & Xu, 2024; Palla, Derényi, Farkas, & Vicsek, 2005; Psorakis, Roberts, Ebden, & Sheldon, 2011; Vieira, Xavier, & Evsukoff, 2020; Xie, Szymanski, & Liu, 2011), which may offer a more organic method to characterize the brain’s spatially distributed and fluid network of complex interactions with an overlapping modular architecture. For example, Wu et al. (2011) applied the clique percolation method (Shen, Cheng, & Guo, 2009) to identify five communities based on gray matter volume correlations, revealing an overlap among well-known functional systems, such as decision-making and emotional processing. Other studies have characterized the hierarchical network architecture of the mouse (Sotiras, Resnick, & Davatzikos, 2015) and macaque (Armas, Knoblauch, Kennedy, & Toroczkai, 2024) structural connectome with algorithms that allow overlapping community assignment, although the overlap itself was an auxiliary point in both studies. By contrast, considerably more work has focused on overlap among functional networks, evaluating functional magnetic resonance imaging (fMRI; Bijsterbosch et al., 2023; Faskowitz, Esfahlani, Jo, Sporns, & Betzel, 2020; Gates, Wood, Hetrick, & Ahn, 2019; Lee, Lina, Gotman, & Grova, 2016; Lei et al., 2024; Li, Gan, & Wang, 2018; Li, Hu, & Wang, 2016; Mirzaei & Soltanian-Zadeh, 2019; Najafi et al., 2016; Zhu et al., 2021) and/or calcium imaging (Vafaii et al., 2024) with a given OCDA to characterize the overlapping community structure in functional networks. Collectively, findings from these studies converge on the view that brain areas spanning multiple structural and/or functional modules enable inter-network communication (“integration”) that still preserves intra-module specialization (“segregation”)—highlighting how OCDAs offer nuanced information about the rich overlapping patterns in brain community structure that can be obfuscated by non-overlapping partitions. Moreover, as explored in Bijsterbosch et al. (2023), an overlapping node may play a non-stationary role, potentially communicating exclusively with one network at a time or consistently serving as a fully integrative bridging node that facilitates information transfer across functionally specialized systems.

Each of these applications of overlapping community detection to neuroimaging data began with a subjective selection of the OCDA from a range of possible alternatives—without clear evidence of the superiority of one approach over the other for the problem at hand. This speaks to a general challenge in applying community detection algorithms: Each algorithm makes different assumptions about how modules and overlapping nodes (which are members of multiple communities) are defined. We know that applying an OCDA to a network will always produce *a result* and that differences in the assumptions between OCDAs yield different assignments of nodes to communities. This multitude of choices for an OCDA algorithm and its parameters thus raises the question: How can we determine which OCDA may exhibit the most accurate and informative decomposition of a given real-world network?

No single algorithm can perform optimally on all possible data (cf. the “no-free-lunch” theorem; Wolpert & Macready, 1997); stated in the present context, no community detection method can work well on all networks (McCarthy, Chen, & Ebner, 2020; Peel, Larremore, & Clauset, 2017). N. R. Smith, Zivich, Frerichs, Moody, and Aiello (2020) developed a “Question-Alignment” approach that tailors the optimal community detection method based on hypothesized community properties, but this requires a priori predictions about community structure without a quantitative benchmark for comparison. Other previous work has benchmarked OCDA performance on typically very large synthetic networks (>1,000 nodes) and/or relatively small empirical networks separately (Aston, Hertzler, & Hu, 2014; Dao, Bothorel, & Lenca, 2020; Hajiabadi, Zare, & Bobarshad, 2017; Hric, Darst, & Fortunato, 2014; Vieira et al., 2020; Xie, Kelley, & Szymanski, 2013; Zhang et al., 2015), although algorithmic performance is largely dependent on network topology. As a result, it is difficult to reliably extrapolate previous benchmark comparisons to a network with different characteristics—size, sparsity, and topology—such as the brain’s structural connectome. Rather than selecting an OCDA subjectively or from a benchmark study that may not generalize to a new complex network type, we need a way to objectively tailor our choice of algorithm to the structure of a given network at hand.

We address this challenge by developing a flexible method for the data-driven selection of an OCDA for a given empirical network. Here, we focus specifically on the right-hemisphere structural connectome as our target empirical network, with the goal of narrowing down the optimal OCDA and its parameter(s) to infer overlapping community structures and interpret their biological significance. Our approach first generates an ensemble of synthetic benchmark networks, with properties derived directly from the target empirical network—the diffusion MRI-derived structural connectome of the right hemisphere from the Human Connectome Project (HCP; Van Essen et al., 2013)—with known “ground-truth” overlapping community assignments, and then compares the performance of a range of OCDAs on these networks. After identifying the top-performing algorithm across the benchmark network ensemble, we then analyze and interpret the resulting overlapping modular decomposition in the context of brain network structure and function. To our knowledge, this is the first work to comprehensively examine overlapping communities in the human brain’s structural connectome using diffusion MRI data. In total, we introduce a novel approach to tailoring OCDA methods to a given real-world network and systematically quantify the overlapping community structure of diverse complex systems.

## METHODS

**Figure 1.**
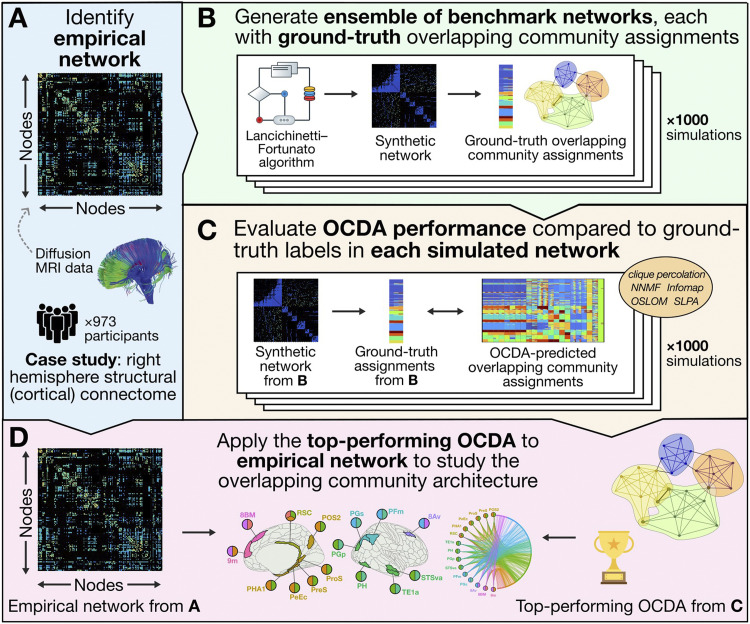
A data-driven method for tailoring overlapping community detection algorithms (OCDAs) to empirical networks. (A) An empirical network is shown as an adjacency matrix. (B) We use a generative network model (Lancichinetti & Fortunato, 2009) to generate an ensemble of networks with known overlapping community assignments, constrained by the properties of the empirical network. (C) We assess the ability of each OCDA (and parameters) to reproduce the ground-truth overlapping community structure of each synthetic network. Here, we compare five OCDAs: clique percolation (Derényi, Palla, & Vicsek, 2005; Palla et al., 2005; Shen et al., 2009), non-negative matrix factorization (NNMF; Psorakis et al., 2011), speaker-listener label propagation algorithm (SLPA; Xie et al., 2011), order statistics local optimization method (OSLOM; Lancichinetti et al., 2011), and Infomap (Rosvall et al., 2009; Rosvall & Bergstrom, 2008), across 22 parameter variations, quantitatively evaluating their ability to reproduce the known community assignments using extended normalized mutual information (ENMI). Algorithms with high ENMI across the ensemble are judged to be most appropriate to infer overlapping community structure in the empirical network. (D) The top-performing OCDA identified in (C) is applied to infer the overlapping community architecture in the empirical network introduced in (A).

Given an empirical network, our goal is to systematically evaluate candidate OCDAs to identify a top-performing candidate for inferring that network’s overlapping community structure. We propose a data-driven approach with two main steps, as depicted schematically in Figure 1: (a) *benchmark calibration* and (b) *performance evaluation*. First, we generate an ensemble of benchmark networks that mimic community structure properties of the empirical network and contain ground-truth module assignments of nodes. Second, for each generated benchmark network in the ensemble, we quantify how well each OCDA identifies the ground-truth overlapping community structure. This highlights algorithms with strong overall performance on the benchmarks, which are then judged to most accurately uncover overlapping community structure, yielding OCDA(s) tailored to the target network.

### Preprocessing Structural Connectome Data

For our target empirical network, we apply the systematic OCDA selection method to structural connectome data collected from 973 participants from the HCP (Van Essen et al., 2013), comprising a subset with complete diffusion MRI data from the broader S1200 release (WU-Minn Consortium Human Connectome Project, 2017). All participants were healthy and aged between 22 and 35 years and provided written informed consent; ethics were approved by the Institutional Review Board of Washington University in St. Louis (Van Essen et al., 2013). Structural MRI volumes were acquired using a 3T Siemens Skyra scanner with a customized head coil (100-mT/m maximum gradient strength and a 32-channel head coil) located at Washington University in St. Louis, MO. Diffusion-weighted images were acquired using a spin-echo echo planar imaging sequence and processed according to the HCP Diffusion pipeline (Glasser et al., 2013), with additional preprocessing using MRtrix3 (Tournier, Calamante, & Connelly, 2012; Tournier et al., 2019) and the FMRIB Software Library (Jenkinson, Beckmann, Behrens, Woolrich, & Smith, 2012). Tractography was conducted in each participant’s structural MRI (T1) space using second-order integration over fiber orientation distributions (iFOD2), a probabilistic algorithm that improves the quality of tract reconstruction in both highly curved and crossing fiber regions (Tournier, Calamante, & Connelly, 2010). Anatomically Constrained Tractography (ACT) and Spherically Informed Filtering of Tractograms (SIFT-2) were applied to the tractography data (R. E. Smith, Tournier, Calamante, & Connelly, 2013) to further improve the biological accuracy of these structural networks. The cortex was parcellated using the 360-region HCP-MMP1 atlas from Glasser et al. (2016), comprising 180 cortical regions per hemisphere.

While there is empirical evidence that the probability for a connection between two regions exponentially decreases with the inter-region distance (Ercsey-Ravasz et al., 2013), the “distance bias” in diffusion preprocessing can further decrease this probability—arising in part from the fact that reconstructing longer tracts introduces more steps for algorithmic errors (Fornito et al., 2016; Sotiropoulos & Zalesky, 2019; Thomas et al., 2014). Given these methodological limitations, we focused on intra-hemispheric connections within a single hemisphere. This allowed us to investigate the connectivity properties within a single contiguous and relatively well-reconstructed cortical sheet, while avoiding analyzing inter-hemispheric connections with poor reliability. This choice is a common one among many studies in structural connectomics (Arnatkeviciute et al., 2021; Betzel et al., 2013; Deco & Kringelbach, 2020; Pang et al., 2023; Vohryzek, Sanz-Perl, Kringelbach, & Deco, 2025). After confirming that the left and right hemispheres exhibit highly similar edge degree, strength, and variance distributions (cf. Supporting Information Figure S1), we selected the right hemisphere for our analysis, focusing on intra-hemispheric connectivity between the 180 right-hemisphere regions in the HCP-MMP1 atlas from Glasser et al. (2016). Moreover, as many studies that have performed (non-overlapping) community detection on whole-brain structural networks reported bilaterally symmetric solutions (Faskowitz, Yan, Zuo, & Sporns, 2018; Lohse, Bassett, Lim, & Carlson, 2014; Sanchez-Rodriguez, Iturria-Medina, Mouches, & Sotero, 2021; Schmidt et al., 2023), we believe the inclusion of the left hemisphere would be unlikely to alter the relative performance trends we observe across algorithms.

We constructed a representative group-level right-hemisphere connectome from the individual-level data using a thresholding approach, which is common practice to minimize contributions of spurious connections (de Reus & van den Heuvel, 2013; Fornito et al., 2016) that are typical in diffusion MRI (Maier-Hein et al., 2017; Thomas et al., 2014). While sparsification of the starting empirical network is not explicitly required for the benchmark network simulation algorithm of Lancichinetti and Fortunato (2009), filtering out noisier edges can improve estimation of parameters used in the algorithm (e.g., community size distributions and the mixing parameter, *μ*). We implemented the consensus-based thresholding approach from Roberts, Perry, Roberts, Mitchell, and Breakspear (2017), retaining only the edges that ranked in the lowest 15% for coefficient of variation (CV) in strength across the entire cohort (*N* = 973). This method retains the most stable edge weights across the entire sample population, with edge weights failing to meet this cutoff threshold set to 0. Of note, one node—*13l*, in the orbitofrontal cortex—exhibited highly variable edge weights (all with CV of 0.3 or higher) and had a total degree of 0 (after applying the 0.15 consensus-based threshold). To prioritize consistency in edge weights for community detection, we thus excluded region *13l* from our analysis. In this group-level connectome, edge weights correspond to the thresholded average number of streamlines between the two regions terminating within a 5-mm radius of each other. Due to the range in streamline count magnitudes across edges, which spanned multiple orders of magnitude, we log-transformed the right-hemisphere edge weights between region–region pairs before further analysis.

### Generating Benchmark Networks

Once we have an empirical network as a starting point (i.e., the preprocessed consensus right-hemisphere structural connectome in this case), the first step in our comparative method involves generating an ensemble of benchmark networks with ground-truth overlapping community assignments, as depicted in Figure 1B. For the subsequent inference to be informative, these generated benchmark networks should have properties as similar as possible to the empirical network. Here, we focus on weighted, undirected networks (corresponding to our intended application of structural brain networks, such as those estimated using diffusion-weighted imaging; Calamante, 2019). We use the stochastic network generation algorithm of Lancichinetti and Fortunato (2009), which—for a given set of parameters that constrain the structural properties of the generated benchmarks—produces weighted networks with overlapping community assignments. Our ability to tune the benchmark networks to match the properties of the target empirical network is controlled by the algorithm parameters, which we describe briefly in the following.

Across a given number of nodes (*N*), Lancichinetti and Fortunato (2009) benchmark networks are assumed to have a power-law degree distribution (*p*_*k*_ ∼ *k*^−*τ*_1_^), with average degree 〈*k*〉 and maximum degree (*k*_max_). Community sizes are sampled from a power-law distribution with exponent *τ*_2_, with a specific minimum and maximum community count along with a set number of overlapping nodes, *O*_*n*_. Edge weights are assigned under the constraint of a power-law relationship between degree (*k*) and strength (*s*) of network nodes, *s* = *k*^*β*^, determined by the parameter *β*. The extent to which a node’s connections (and their weights) are contained within its own communities (or spread across other communities) is controlled through two mixing parameters: (a) *μ*_*t*_, the average fraction of a node’s edges that are external to the communities it belongs to, and (b) *μ*_*w*_, the average fraction of a node’s aggregate edge weights external to the communities to which it belongs. Given these parameters, benchmark networks are generated by forming links between nodes within and outside their assigned communities across iterative network rewirings.

To generate benchmark networks with properties that match the empirical network as closely as possible, we require a systematic method to fit the generative parameters of the Lancichinetti and Fortunato (2009) algorithm. Some parameters that control the basic connectivity properties of the network (*N*, 〈*k*〉, and *k*_max_) were computed directly from the empirical network or—in the case of *τ*_1_ and *β*—were estimated from power-law fitting algorithms (Clauset, Shalizi, & Newman, 2009). Other parameters governing the community structure (e.g., number and size of communities and overlapping nodes) cannot be directly inferred from the network, but instead require subjective judgment about the type and resolution of community structure that is most appropriate for the application of interest. In the absence of ground-truth parameter values based on clear domain knowledge (or other priors on the community structure), we sampled these parameters from uniform distributions across reasonable ranges. Specifically, we allowed communities to take any size (from 1 − *N* nodes), and sampled the power-law distribution exponent, *τ*_2_ ∼ *U*(2, 4), and the number of overlapping nodes, *O*_*n*_ ∼ *U*(0.1*N*, 0.2*N*), from uniform distributions. To ensure that connectivity was predominantly within communities, mixing parameters were sampled in ranges: *μ*_*t*_ ∼ *U*(0.2, 0.4) and *μ*_*w*_ ∼ *U*(0.2, 0.4). Using this combination of fixed parameters estimated from the network, (*N*, 〈*k*〉, *k*_max_, *τ*_1_, *β*), and randomly sampled parameters representing reasonable ranges of the overlapping modular structure of interest, (*τ*_2_, *O*_*n*_, *μ*_*t*_, *μ*_*w*_), we generated an ensemble of 1,000 benchmark networks, each resulting from a sampled parameter vector.

The Lancichinetti and Fortunato (2009) generative network model makes assumptions about the data that may not hold in all cases; for example, the power-law form of degree distribution that this model assumes will not always be a good representation of real network data. As alternative models are developed in the future that can better fit the key structural properties of a real network, these could be substituted into this benchmark generation step to improve the representativeness of the simulated benchmark networks to the real network data.

### OCDAs

Once we have generated an ensemble of benchmarks that resemble the empirical network (Figure 1B), we next compare the performance of OCDAs in reproducing the known community structure (Figure 1C). Here, we investigated five different OCDAs, each with various parameter options and delineated briefly below.

*Clique percolation* (Derényi et al., 2005; Palla et al., 2005) generates overlapping communities by clustering a maximal clique network and then projecting this hard partition of maximal cliques back to an overlapping partition of nodes. We used the algorithmic implementation of clique percolation developed in Shen et al. (2009), which introduces a “quality of network coverage” measure (*Q*_*c*_) that is optimized based on the maximal clique view. Specifically, a clique is a complete subgraph, and maximal cliques are not subgraphs of any other cliques in a graph. The method first generates a new network, *G*′, in which each node is the maximal clique of the original network, *G*, and then computes a hard partition of this new network. As some nodes may be members of multiple cliques, this yields overlapping community assignments in the original network; importantly, the implementation in Shen et al. (2009) ensures that all nodes are assigned to at least one clique such that there are no isolated nodes, which is a departure from the original method in Derényi et al. (2005) and the subsequent extension to incorporate weighted edges in Farkas, Ábel, Palla, and Vicsek (2007). The algorithm includes a parameter, *k*, that sets the minimum size of maximal cliques to be mapped in the new network, *G*′. Here, we compare seven possible values of this clique size parameter, *k* = 3, 4, …, 9, labeling the resulting algorithms, correspondingly, as: Clique_3, …, Clique_9.

*Non-negative matrix factorization* (*NNMF*; Psorakis et al., 2011) uses a Bayesian NNMF model to assign a participation score to each node for each community, which is based on the interaction between that node with other members of the community. NNMF assigns every node a soft membership distribution across communities that can be interpreted as a membership probability of each node to each community. Specifically, we row-normalized the non-negative matrix *W* from the NNMF decomposition *X* ≈ *WH*, yielding the matrix *P*, where the probability of node *i* participating in community *k* is given as

$$P_{ik}=\frac{W_{ik}}{\sum_{k^{\prime }=1}^{i}W_{ik^{\prime }}}.$$ (1)

To convert this set of continuous community membership probabilities into a set of discrete labels defining the set of community memberships for a given node, we applied a threshold, *p*_th_. That is, the node *i* is considered a member of community *k* if its community membership probability *P*_*ik*_ ≥ *p*_th_. We compared four thresholds, *p* = 0.1, 0.2, 0.3, 0.4, and labeled the resulting algorithms as NNMF_10, NNMF_20, NNMF_30, and NNMF_40, respectively. While the theoretical space of solutions in NNMF is non-unique, the combination of row normalization and Bayesian regularization in the method from Psorakis et al. (2011) promotes consistent and interpretable membership distributions across networks.

*Order statistics local optimization method* (*OSLOM*; Lancichinetti et al., 2011) is a local method that attempts to find and modify the statistical significance of clusters relative to a global null model in which there is no community structure (the configuration model; Molloy & Reed, 1995) by adding or removing neighboring nodes. Thus, OSLOM yields clusters that are statistically unlikely to be found in an equivalent random graph with the same degree sequence; the set of individual clusters found in a network may overlap. In adjusting the significance level, *P*, from which to assess whether to make local changes to a cluster, the resulting size of clusters is changed; low values of *P* yield fewer, larger clusters. We compared a range of 10 values *P* = 0.1, 0.2, …, 1.0, denoted as OSLOM_10, OSLOM_20, …, OSLOM_100, respectively.

*Speaker-listener label propagation algorithm* (*SLPA*; Xie et al., 2011) is an extension of the label propagation algorithm (LPA; Raghavan, Albert, & Kumara, 2007) to the overlapping setting. In LPA, the community assignments of individual nodes are updated iteratively based on the majority label possessed by their immediate neighbors. In the overlapping generalization, SLPA, nodes are not forced to take a single label, but can hold the memory of previously observed labels; the more frequently a node has observed a node label, the more likely it is to then propagate it to its neighbors. After some maximum number of iterations of label propagation, nodes are assigned to communities based on the most frequently observed labels in their memories. Once communities are identified, SLPA removes labels observed less than the fraction *r*. While *r* values in the range 0.01–0.45 have been used previously (Asadi & Ghaderi, 2018; Kuzmin, Chen, & Szymanski, 2015; Larasati & Surjandari, 2017; Rajeh & Cherifi, 2023), here, we set *r* = 0.09 as it sat within the range of thresholds yielding convergent results in the original SLPA publication (Xie et al., 2011). Xie et al. (2011) note that the set threshold is exclusively for postprocessing (i.e., refining communities after they have been defined), such that the SLPA dynamics are determined solely by network topology and employed interaction rules. We denote this method as SLPA.

*Infomap* (Rosvall et al., 2009; Rosvall & Bergstrom, 2008; Viamontes Esquivel & Rosvall, 2011) is an information-theoretic method operating under the principle that network communities can be identified by compressing the description of the probability flow of random walks. It solves the map equation, which quantifies the description length of a random walker’s trajectory through the network, by balancing the information required to describe movements between communities and that needed to move within communities. Infomap involves the construction of a “codebook,” meaning a set of labels (or “codes”) assigned to different parts of the network that efficiently capture the trajectories of the random walker within the network. This method was extended to capture overlapping nodes using a generalized map equation that allows these codebooks to allow multiple modules assigned to a single node (Viamontes Esquivel & Rosvall, 2011). We denote this algorithm as Infomap.

In our benchmark comparison procedure, outlined in the following sections, the five OCDA methods and corresponding parameter combinations yielded a total of 22 specific OCDAs to evaluate. One important distinction between OCDA types rests on their treatment of edge weights. Some algorithms, such as clique percolation and SLPA, require the input network to be binarized—effectively representing the network as a topology of connections, without consideration of connection strengths. This binarization can result in a loss of information for a network where edge weights carry meaningful structural and/or functional significance (Lu, Sun, Wen, Cao, & La Porta, 2015), as in brain connectivity data. On the other hand, algorithms like OSLOM, NNMF, and Infomap are inherently designed to incorporate the graded structure of edge weights directly into the process of community detection. This methodological divergence has important implications for benchmarking, such that comparing outputs from binarized versus weighting-sensitive algorithms requires careful consideration of how (and if so, the extent to which) the usage of edge weights might affect algorithm performance. We chose to include both types of algorithms in our analysis for a representative cross-section of common OCDAs used across disciplines. To bolster comparability, we opted for implementations with only one (if any) user-defined parameter to systematically vary such that all other conditions are fixed per algorithm. While edge-weight sensitivity may confer a potential advantage in networks with informative edge weight distributions, our goal is to compare all methods under conditions matched as closely as possible (manipulating only the key parameter per algorithm; e.g., *k* in clique percolation) to isolate algorithm-specific differences in overlapping community detection performance.

### Performance Evaluation

After applying each OCDA to infer the overlapping community structure in each benchmark network, we next assessed how well each algorithm reproduces the ground-truth overlapping community assignment, as depicted in Figure 1C. Quantifying the relative performance of OCDAs is challenging, although several measures have been developed to capture different aspects of the goodness-of-fit relative to a ground-truth community decomposition for a synthetic benchmark network (or ensemble of networks, in our case). Here, we used extended normalized mutual information (ENMI) to quantify the overall similarity of two overlapping community assignments. ENMI is an extension of normalized mutual information (NMI), which was introduced in Danon, Diaz-Guilera, Duch, and Arenas (2005) (and further discussed in Newman, Cantwell, & Young, 2020). NMI is defined as

$$I_{\text{norm}}\left(X;Y\right)=\frac{I\left(X;Y\right)}{\left[H\left(X\right)+H\left(Y\right)\right]/2},$$ (2)

where *I*(*X*; *Y*) is the mutual information (MacKay, 2003) between the random variables *X* and *Y*. NMI captures the amount of information shared between *X* and *Y*, quantified as the reduction in uncertainty in *X* while observing *Y* (and vice versa; this is a symmetric measure). For a non-overlapping community assignment, *X* and *Y* for a given node *i* are coded as integers, that is, *x*_*i*_ = 1, 2, …, ∣*C*_*X*_∣, up to the total number of communities in the first partition ∣*C*_*X*_∣, and similarly for *y*_*i*_.

In the case of an overlapping decomposition, multiple community assignments per node can be captured using a binary encoding, with (**x**_*i*_)_*k*_ = 1 if node *i* is a member of community *k*, and (**x**_*i*_)_*k*_ = 0 otherwise. In this case, for each node *i*, each community *k* can then be considered as a random variable, for example, as *X*_*k*_ = (**X**)_*k*_, with random variables for all *k* communities being captured as **X**. Probabilities *P*(*X*_*k*_ = 1) and *P*(*X*_*k*_ = 0), and similarly for *Y*_*l*_, can be estimated from a given overlapping modular decomposition, allowing information-theoretic computations to be performed on the resulting collections of binary random variables **X** and **Y**. Lancichinetti, Fortunato, and Kertész (2009) adapted NMI to apply to this overlapping setting, yielding ENMI, as outlined in Appendix B of Lancichinetti et al. (2009). ENMI ranges from 0 to 1, and given our setting of comparing a ground-truth overlapping community assignment **X** with an algorithm-predicted assignment **Y**, a larger ENMI value indicates superior recovery of the underlying overlapping community structure in the benchmark network.

ENMI is frequently implemented as a proxy for the goodness of fit with which an algorithm infers the ground-truth overlapping community structure in a given network (Asmi, Lotfi, & Abarda, 2022; He, Jin, Chen, & Zhang, 2015; Kim & Jeong, 2011; Sun et al., 2017; Xie & Szymanski, 2012), including the study by Xie et al. (2011), which introduced the SLPA method included in our comparisons. Lancichinetti et al. (2009) demonstrated that ENMI increases monotonically as the community structure becomes more pronounced (based on varying the mixing parameter in the same generative benchmark network algorithm employed in this study). Moreover, while it is not straightforward to evaluate the “accuracy” of specific node-to-module assignments in a label-matched manner across algorithms, ENMI captures the overall match to ground-truth community structure in a label-invariant manner. Together, ENMI is theoretically suitable for the multi-membership nature of overlapping communities, and its implementation makes it flexible for cases where the algorithm introduces extra communities beyond the number of ground-truth communities.

OCDAs introduce additional algorithmic complexity and computational burdens beyond those of a non-overlapping option, like the Louvain method (Blondel, Guillaume, Lambiotte, & Lefebvre, 2008), which is the most widely used community detection method in network neuroscience (Betzel, 2023). In order to evaluate whether an OCDA better recovers community structure in a network with known ground-truth overlaps than a simpler non-overlapping method, we additionally applied the Louvain (non-overlapping) community detection algorithm to each network in the benchmark ensemble. This allowed us to compute the ENMI between the resulting Louvain decomposition and the ground-truth community structure in order to compare if, and by how much, an OCDA improves community detection in simulated networks resembling the brain’s right-hemisphere cortical connectome. We then compare the computational runtime for each method across the 1,000 simulated networks in the benchmark ensemble to evaluate performance–computational burden tradeoffs across the methods (including Louvain).

Finally, in order to evaluate how well each OCDA correctly identifies specific overlapping nodes in the benchmark ensemble, we compared the sensitivity and specificity for the ground-truth nodes in each network. In both cases, we binarized all 180 nodes (i.e., regions in the right cortex) according to both the ground-truth assignments (*X*) and the algorithm-predicted assignments (*Y*), assigning a value of 1 only if that node belongs to two or more communities. This allowed us to construct a confusion matrix, where “positive” corresponds to overlapping assignment and “negative” corresponds to single-community assignment. We then computed the sensitivity and specificity for each OCDA and each simulated network in the benchmark ensemble. These metrics complement the ENMI, which summarizes across all 180 nodes regardless of overlapping status, to focus specifically on the recovery of ground-truth overlapping node identity. A high sensitivity means that the algorithm correctly identified many of the overlapping nodes in the network, while a high specificity means that the algorithm did not assign many non-overlapping nodes to multiple communities.

### Applying OSLOM to the Empirical Structural Human Connectome

Since OSLOM was identified as the top-performing OCDA in the benchmark ensemble analysis, we further refined its threshold parameter (*P*) for the applied case study analysis with the empirical right-hemisphere cortical connectome. We compared modular decomposition at each *P* = 0.01, 0.02, …, 1 to identify a suitable range of *P* values, across which OSLOM identified a consistent number of modules and overlapping nodes. Our empirical selection of a representative *P*-value threshold for application to the observed right-hemisphere structural connectome is guided here by decomposition stability, rather than in-sample performance, precisely to minimize the risk of overfitting. Importantly, OSLOM is a stochastic algorithm, such that the resulting decomposition can vary depending on the user-supplied seed for the pseudo-random number generator (which determines which nodes are initially selected as the basis for community evaluation; Lancichinetti et al., 2011). Most prior applications of OSLOM to interdisciplinary real-world problems that specify *P* and algorithm hyperparameters do not specify the random seed used to generate their results (in which case, the seed is automatically generated based on the time at analysis, down to the microsecond; De Deyne, Verheyen, Perfors, & Navarro, 2015; Esteve-Altava, 2017). Bruner, Esteve-Altava, and Rasskin-Gutman (2019) used the singular pre-specified seed of 73, and Acman, van Dorp, Santini, and Balloux (2020) examined a handful of random seeds (1, 5, 42, 93, 212) before manually selecting results obtained with an individual seed (42). Gates et al. (2019) did not specify whether seeds were set, but they applied OSLOM to the functional brain connectome 10 times per participant, deriving an “element-centric similarity matrix” based on the similarity of individual OSLOM decompositions between participants.

Recognizing that the robustness of a particular decomposition is essential for inferring generalizable biological significance in the human connectome, we systematically evaluated the variability of OSLOM outputs in the fixed-parameter setting across different random initialization seeds. We verified that OSLOM produced deterministic outputs for a given initialization seed and *P*-value threshold combination, mitigating concerns about internal stochasticity. For each threshold between 0.01 and 1, we ran OSLOM using 100 different initialization seeds (setting the seed to each of the integers from 1 to 100) and examined the average number of modules and number of overlapping nodes across thresholds. After narrowing the *P*-value range down to *P* = 0.15, 0.16, …, 0.35 based on the stability in the number of modules and overlapping nodes (averaged across seeds; cf. Supporting Information Figures S2A–S2B), we then selected a final value of *P* = 0.3 as an intermediate value that yielded top performance in the simulated benchmark analysis.

Having selected *P* = 0.3, we next sought to identify a particular decomposition from the 100 realizations (corresponding to the 100 initialization seeds) that is most similar to (i.e., most “central”) all other decompositions—analogous to selecting a Fréchet mean in a distance-based framework. We quantified similarity in modular decomposition between each pair of seeds using the ENMI (Supporting Information Figure S2C; cf. Performance Evaluation section) for consistency in the comparison measure used to evaluate each OCDA against each simulated network in the benchmark ensemble. We additionally constrained our search to seeds that yielded module sizes between 10 and 60 nodes (inclusively) in order to focus on biologically interpretable region groups. This approach favors the selection of a solution that is most stable (i.e., consistent) across initialization, providing a single representative decomposition that best captures the consensus structure detected by the algorithm (in this case, OSLOM). This similarity analysis identified Seed 61 as the most representative, exhibiting a mean ENMI of 0.64 ± 0.11 with all other seeds; for comparison, the mean ENMI across all other seeds was 0.59 ± 0.10. Of note, seeds with higher average ENMI values (including Seed 61) also exhibited greater variance in ENMI values; this relationship is depicted as a scatterplot in Supporting Information Figure S3A. Although we did not explicitly penalize for variance in our seed selection, the tight coupling between mean and median ENMI values shown in Supporting Information Figure S3B supports its use as a proxy for “representativeness.” All subsequent analyses in the empirical right-hemisphere cortical connectome case study were conducted using the modular decomposition derived from this seed–parameter combination. Together, while stochasticity in community detection algorithms is challenging, we developed and implemented a systematic approach from which we selected a representative result from OSLOM to subsequently analyze.

### Comparing Overlapping Nodes With Biologically Relevant Properties

In order to investigate various aspects of how the overlapping community structure relates to the putative functional hierarchy in the cortex, we used principal gradients (PGs) of functional connectivity (Margulies et al., 2016). We obtained pre-processed maps of the first and second PGs (PG1 and PG2, respectively) from the *neuromaps* Python package (Version 0.0.5; Markello et al., 2022). After transforming the reference maps into fsaverage surface space, we analyzed the vertex-wise values for PG1 and PG2 for each of the 180 regions in the HCP-MMP1 parcellation (Glasser et al., 2016). We summarized the mean values across vertices to obtain region-averaged values for PG1 and PG2, respectively.

### Comparing Overlapping and Non-Overlapping Modular Partitions

To clarify whether the top-performing OCDA can provide additional and unique information beyond that of a non-overlapping partition, we compared network properties from the two modular decomposition types. For a representative non-overlapping partition, we applied Louvain community detection using the method from Rubinov and Sporns (2010), first tuning the resolution parameter *γ* to yield relatively consistent decompositions across 100 iterations, each with the seed set to an integer from 1, 2, …, 100 (as with OSLOM). After sweeping across *γ* values ranging from 0.5 ≤ *γ* ≤ 1.5, we selected *γ* = 1 (which happens to be the default in the Brain Connectivity Toolbox; Rubinov & Sporns, 2010), as this forms an elbow in the number of communities with lower variance across seeds than higher *γ* values (as plotted in Supporting Information Figure S4A). We selected a representative seed (98) based on mean NMI with all other seeds (total of 100, as shown in a heatmap in Supporting Information Figure S4B), which yielded a final six-community decomposition—projected onto the right-hemisphere cortical surface in Supporting Information Figure S4C. As a robustness test, we also compared results using *γ* = 1.5 to enforce a seven-module (non-overlapping) decomposition for the direct comparison of seven communities between OSLOM and Louvain, with the results shown in Supporting Information Figures S4D–S4G.

We computed two frequently used nodal measures from the Louvain decomposition using the Python implementation of the Brain Connectivity Toolbox (Rubinov & Sporns, 2010; bctpy, https://github.com/aestrivex/bctpy): (a) within-module strength (*z*-scored), *z*, which measures how strongly a node is connected to others within its own community, and (b) participation coefficient, *P*, which measures the diversity of a node’s connections across network modules (Cajic et al., 2024; Fornito et al., 2016; Guimerà & Nunes Amaral, 2005). To understand the relative position of each OSLOM-defined overlapping node within the non-overlapping Louvain partition, we plotted all nodes in *z* − *P* space, following the characterization in Guimerà and Nunes Amaral (2005). For direct comparison with the top-performing OCDA, we adapted the algorithm for *P* to allow each overlapping node to be assigned to multiple modules.

### Data Visualization and Code Availability

The overlapping community decomposition was visualized within the parcellated cortical surface using the *ggseg* package in R (Mowinckel & Vidal-Piñeiro, 2020). We used the *NetworkX* package (Hagberg, Swart, & Schult, 2008) in Python to visualize community structure with a “circos” layout and an undirected graph network layout in Figure 4B. Raincloud plots are visualized using the “see” (Lüdecke et al., 2021) and “ggplot2” (Wickham, 2016) packages in R. An open and extendable MATLAB implementation of our methodology is available on GitHub^1^, which also includes all codes to reproduce our main results in a combination of MATLAB (and R, for visualization purposes only). The methods schematic in Figure 1 includes images provided under CC BY 2.0 by Eunice Kennedy Shriver National Institute of Child Health and Human Development (flickr.com/photos/nichd/16672073333) and CC0 by Wikimedia Commons (https://commons.wikimedia.org/wiki/File:Illustration_of_overlapping_communities.svg, https://upload.wikimedia.org/wikipedia/commons/a/a6/Trophy_Flat_Icon.svg).

## RESULTS

Here, we apply our data-driven algorithm selection method to characterize the overlapping modular structure of the right cortical connectome, derived from 973 individuals in the HCP dataset (Van Essen et al., 2013). As depicted schematically in Figure 1, our systematic approach generates an ensemble of benchmark networks with properties that mirror the target empirical connectome, with a ground-truth overlapping structure that enables selection of the top-performing OCDA. After identifying OSLOM as the top-performing algorithm across the benchmark ensemble, we then analyze the overlapping community structure inferred by this method, comparing the resulting decomposition with functional properties to demonstrate the unique insights beyond those that can be inferred from a traditional non-overlapping partition.

### The Connectome-Tailored Benchmark Network Ensemble Highlights OSLOM as the Top-Performing Algorithm

**Figure 2.**
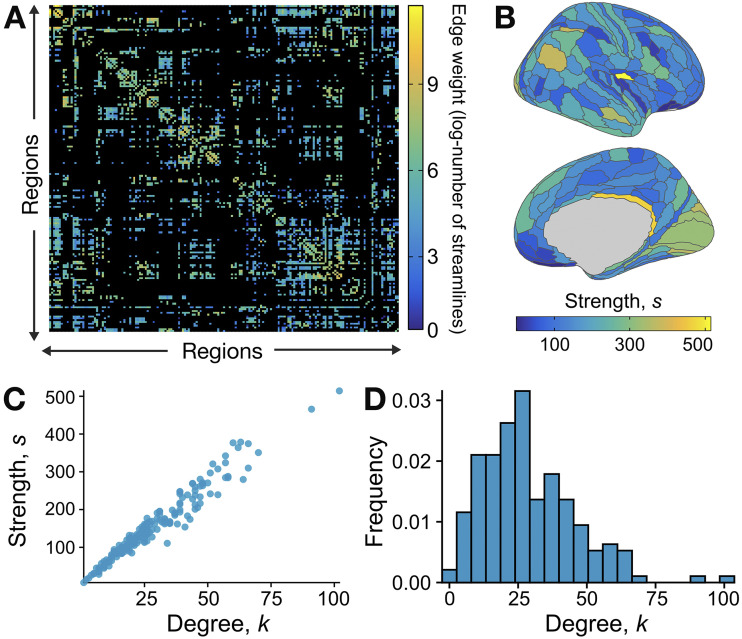
Network properties of the group-level structural connectome of the right hemisphere of the human cortex. (A) The empirical structural connectome of the right cortical hemisphere is shown as a weighted 180 × 180 adjacency matrix, with colors representing the log-transformed number of streamlines (connections) between nodes. (B) The strength, *s*, is computed for each region (as the sum of all log-transformed edge weights) and projected on the cortical surface for the right hemisphere. (C) Plotting node strength (*s*) versus node degree (*k*, the sum of binarized connections) demonstrates that *s* ∝ *k*—enabling the inference that *s* ∝ *k*^*β*^, with *β* = 1, for Lancichinetti and Fortunato (2009) benchmark network generation. (D) The frequency distribution for degree (*k*) values across nodes, which does *not* exhibit power-law scaling.

In order to tune parameter values for the generative network algorithm of Lancichinetti and Fortunato (2009), we first examined key network properties of the empirical human right-hemisphere structural connectome. We constrained our analysis to intra-hemispheric connections in the right hemisphere, confirming that the left and right hemispheres exhibited highly similar edge degree, strength, and variance distributions (cf. Supporting Information Figure S1). For the 180-node right-hemisphere human structural connectome, shown in Figures 2A–2B, we followed the general procedure depicted in Figure 1. We first needed to set the benchmark-generation parameters, (*N*, 〈*k*〉, *k*_max_, *τ*_1_, *τ*_2_, *β*, *O*_*n*_, *μ*_*t*_, *μ*_*w*_), to match the data, as described in the Generating Benchmark Networks section. Basic network parameters were computed directly from the network: *N* = 180, 〈*k*〉 = 29, and *k*_max_ = 102. Plotting node degree, *k*, against node strength, *s*, shown in Figure 2C, revealed *s* ∝ *k*, allowing us to identify the exponent in *s* ∝ *k*^*β*^, as *β* = 1. A limitation of the Lancichinetti and Fortunato (2009) algorithm is its assumption of a power-law degree distribution, *p*_*k*_ ∼ *k*^−*τ*_1_^, which, as shown in Figure 2D, does not hold for this structural connectome. Of note, deviations from power-law distributions have been reported in diverse connectomic datasets and preprocessing paradigms (Gajwani et al., 2023; Gastner & Ódor, 2016; Hagmann et al., 2008), suggesting that this is not unique to the dataset and/or analytical choices implemented here. We nevertheless set *τ* as the best power-law fit to this distribution, as *τ*_1_ = 2.

Sampling from all other parameters across appropriate ranges (using uniform distributions, as described in the Generating Benchmark Networks section), we generated an ensemble of 1,000 right-hemisphere structural connectome benchmark networks, each with a ground-truth overlapping community assignment. The benchmark ensemble was generated on a MacBook Air with an Apple M2 chip and 16 GB RAM, and with the above-described parameter configurations; the 1,000 networks were generated and saved in 224 s (3.7 min). We evaluated the performance of 22 OCDAs (based on five representative algorithms) on each generated benchmark network using the ENMI metric (cf. Performance Evaluation section). For each simulated network, we fit each of the 22 OCDAs comprising five core methods (Infomap, SLPA, OSLOM, clique percolation, and NNMF), along with the Louvain method (Blondel et al., 2008) as a non-overlapping baseline comparison method. We compare the computational runtime required for each algorithm group in Table 1, finding that among the OCDAs—all of which took at least one order of magnitude longer than Louvain (0.002 ± 0.001 s per 180 × 180 network; median ± interquartile range, IQR)—SLPA was the fastest (0.04 ± 0.01 s), while OSLOM was the slowest (12 ± 6 s).

**Table 1.** Computational runtime for the OCDAs (and Louvain as a baseline) across the 1,000 networks in the benchmark ensemble

**Method**	**Median runtime (s)**	**IQR runtime (s)**
Louvain	0.002	0.001
SLPA	0.04	0.01
Infomap	0.4	0.1
Clique percolation	0.7	0.4
NNMF	1.1	0.1
OSLOM	12	6

All analyses were computed sequentially on a MacBook Air with an M2 silicon chip and 16 GB of RAM. Methods are sorted in order of median runtime from smallest to largest. IQR = interquartile range.

**Figure 3.**
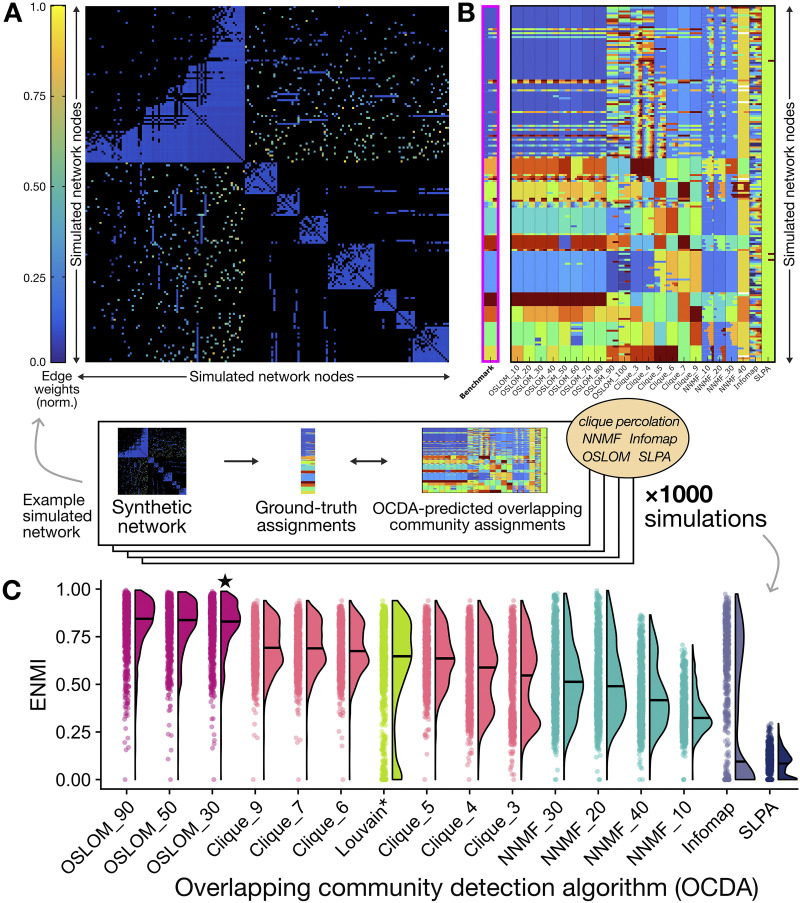
Overlapping community structure predicted by OCDAs (across 22 different combinations of parameters for five types of OCDA methods) on a representative connectome-like benchmark network. (A) A representative simulated network from the benchmark ensemble of 1,000 networks, composed of 180 × 180 nodes representing the right-hemisphere HCP-MMP1 regions (Glasser et al., 2016) as with the empirical structural connectome in Figure 2A. Regions are sorted according to the “Benchmark” (i.e., ground-truth) community assignment, and cells are colored according to the (simulated) connection strength, normalized to the unit interval [0, 1] for visualization. (B) The “Benchmark” column (highlighted in pink with bold text) represents the ground-truth community structure for the network depicted in (A), in which each of the 180 nodes is assigned to one or more communities (represented by colors). Nodes belonging to the same community are assigned the same color, and overlapping nodes are assigned multiple colors. The other 22 columns on the right depict predictions of the overlapping community structure from each OCDA. Note that colors designate different communities within each OCDA column, but individual colors are not directly comparable between OCDA results (i.e., between columns). (C) For each algorithm, ENMI performance distributions are shown as raincloud plots across the generated ensemble of 1,000 benchmark networks. Algorithms are sorted by mean ENMI across the ensemble (shown as black horizontal lines), and algorithms are color-coded as 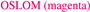, 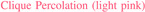, 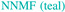, 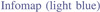, and 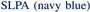. ENMI values are also shown for the 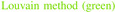 as a baseline non-overlapping method for comparison. Note that OSLOM results are shown here for three selected resolutions (*P* = 0.3, 0.5, 0.9); ENMI distributions are similar for other values of *P*.

We schematically depict our process for evaluating the decompositions generated by each OCDA across the 1,000 networks simulated with the Lancichinetti and Fortunato (2009) method in Figure 3A, highlighting one representative benchmark network for illustrative purposes. The “ground-truth” community structure and overlapping community assignments are shown in Figure 3B, along with those predicted by each of the 22 OCDAs. Visually, we see that variations of the OSLOM method best recapitulate the known overlapping community structure of this representative benchmark, although clique percolation results (with larger *k*-clique sizes) also exhibit similar structure to the ground-truth decomposition. To summarize across all 1,000 generated benchmark networks, in Figure 3C, we show the ENMI distributions as raincloud plots for each OCDA configuration (excluding OSLOM with parameter settings that yielded redundant decompositions). We also included the ENMI distribution for the Louvain method for comparative purposes, with results also summarized in Table 2. The three OSLOM algorithms yielded the highest median ENMI values (0.83–0.84, IQR 0.15–0.16), indicating that OSLOM best uncovered the community structure across human right-hemisphere structural connectome-like benchmark networks out of the methods compared here (cf. Supporting Information Figure S5). Of note, all parameter configurations of OSLOM yielded higher median ENMI values than the Louvain partition (0.65, IQR 0.31), collectively supporting its utility despite the higher computational burden compared with other methods. Clique percolation algorithms exhibited the second-highest median ENMI values (0.55–0.69, IQR 0.19–0.37), although only the larger cliques (*k* ≥ 6) yielded higher median ENMI values than the Louvain decomposition. As noted in the Methods section (cf. Performance Evaluation section), clique percolation binarizes edge weights whereas OSLOM retains edge connection strengths, which may be a contributing factor to the performance differences between the two algorithms. Within NNMF, maximal ENMI values were achieved with probability thresholds (*p*) > 0.1, while Infomap and SLPA exhibited generally weaker performance; all three general OCDA classes yielded lower median ENMI values than the Louvain method.

**Table 2.** ENMI for the OCDAs (and Louvain as a baseline) across the 1,000 networks in the benchmark ensemble

**Method**	**Median ENMI**	**IQR for ENMI**
OSLOM_90	0.84	0.15
OSLOM_80	0.84	0.15
OSLOM_60	0.84	0.16
OSLOM_70	0.84	0.15
OSLOM_50	0.84	0.16
OSLOM_40	0.83	0.15
OSLOM_30	0.83	0.15
OSLOM_20	0.82	0.15
OSLOM_10	0.82	0.17
OSLOM_100	0.79	0.17
Clique_9	0.69	0.19
Clique_7	0.69	0.19
Clique_6	0.68	0.19
Louvain	0.65	0.31
Clique_5	0.64	0.21
Clique_4	0.59	0.32
Clique_3	0.55	0.37
NNMF_30	0.51	0.27
NNMF_20	0.49	0.29
NNMF_40	0.42	0.23
NNMF_10	0.32	0.12
Infomap	0.10	0.70
SLPA	0.08	0.13

In terms of recovering overlapping nodes specifically, we also evaluated the sensitivity and specificity of algorithm-predicted overlapping nodes relative to the ground truth in each simulated network. As shown in Supporting Information Figure S6, NNMF with different thresholds yielded the highest overall sensitivity (meaning that it recovered the most ground-truth overlapping nodes out of all evaluated algorithms), but specificity values were considerably lower than for other algorithms—indicating that NNMF generally predicts more nodes to be overlapping than are actually overlapping. Moreover, NNMF also yielded overall considerably lower ENMI values compared with OSLOM, clique percolation, and even the non-overlapping Louvain decomposition, suggesting that its strong performance in identifying overlapping nodes does not directly generalize to its overall network community structure inference. OSLOM consistently yielded the second-highest range of sensitivity values (31.6%–60.9%) across *P* thresholds, while maintaining 100% specificity (with the exception of OSLOM-100, which exhibited a specificity of 96.8 ± 5.2%). The other three OCDA classes—clique percolation, Infomap, and SLPA—all exhibited near-perfect specificity (all >97%) yet very low sensitivity to ground-truth overlapping nodes (all <17%). Together, the benchmark ensemble analysis suggests that OSLOM best recovers the overlapping community structure of the human (right-hemisphere) structural connectome out of the evaluated OCDAs, in terms of overall community identification (ENMI analysis) as well as identification of specific overlapping nodes (sensitivity/specificity analysis). Given these results, we progressed with OSLOM as the preferred OCDA to study the structural architecture of this intra-hemispheric cortical connectome dataset.

### Overlapping Community Detection Reveals Regions That Bridge Multiple Networks and Sit High in the Cortical Hierarchy

Having identified OSLOM as the top-performing algorithm for uncovering the overlapping community structure in our right-hemisphere structural connectome dataset, we progressed with this algorithm to characterize its overlapping community structure. As described in the Methods section (Applying OSLOM to the Empirical Structural Connectome section), we performed a more comprehensive parameter sweep (across *P*-value thresholds in finer increments of 0.01 across 100 initialization sweeps) and identified a plateau in the number of modules detected (six to seven) and the number of overlapping nodes detected (14 to 15) between 0.15 ≤ *P* ≤ 0.35 (cf. Supporting Information Figures S2A, S2B). We selected an intermediate value within the stable plateau of *P* = 0.3, which also yielded among the highest ENMI with the ground-truth overlapping community structure in the benchmark analysis (cf. Supporting Information Figure S2C). After examining pairwise ENMI values across initialization seeds to identify an optimally representative decomposition, we selected seed 61 (highlighted in the ENMI heatmap in Supporting Information Figure S2), which exhibited a mean ENMI of 0.64 ± 0.11 with all other examined seeds at *P* = 0.3. This selection of parameters is referred to hereafter as OSLOM_30.

**Figure 4.**
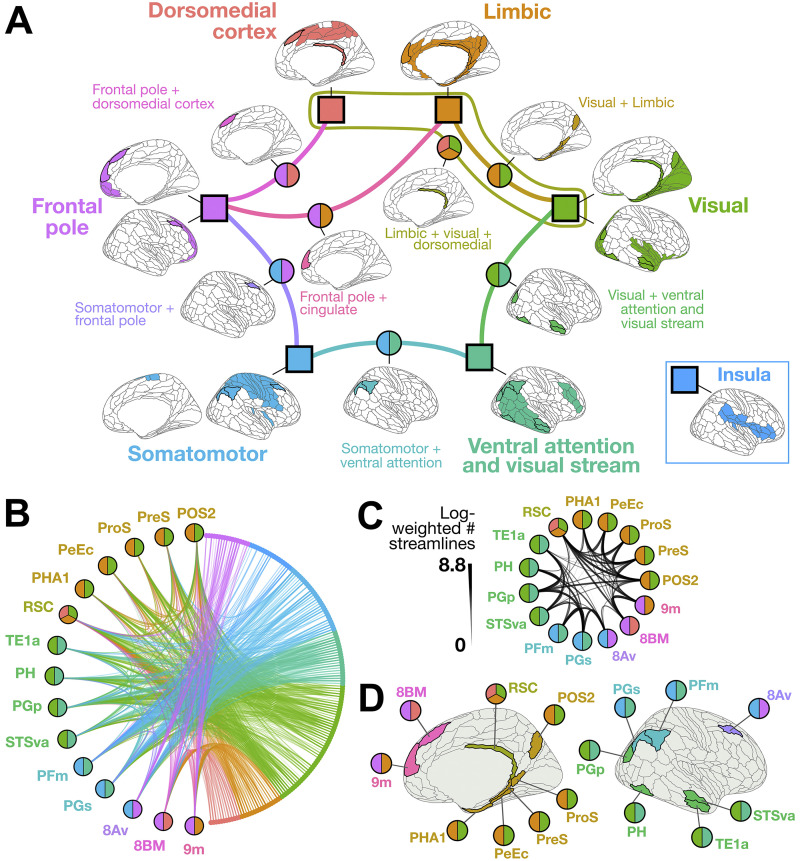
The overlapping community structure of the human structural connectome (right hemisphere) shows anatomical specificity and cross-network structural connectivity. (A) The OSLOM_30 algorithm identified seven overlapping communities of the human structural connectome, which are represented with square nodes and corresponding brain maps with the 180-region parcellation from Glasser et al. (2016). Regions colored in a given map at each node were assigned to the corresponding module (and potentially to another module as well). The edges and their corresponding brain maps indicate regions with overlapping community assignments to two or more communities. Overall, the resulting communities are anatomically localized to the following regions: 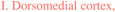 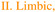 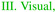 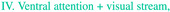 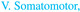 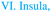 and 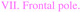 Note that the “Insula” module is positioned to the side as it does not share any overlapping connections to any of the other communities. (B) In this connectogram, every node is plotted on the edge of the circle, ordered by assigned structural module. Lines connecting the nodes indicate the connectivity between the pair of regions, with width and transparency showing the log-transformed streamline count. Fifteen brain regions were assigned to two or more different communities (“overlapping regions”), distinguished on the left of the plot with two- or three-tone circles used to indicate the networks bridged by the corresponding region. The full structural connectome is summarized between these 15 overlapping regions (left) and the rest of the brain (right), with the latter colored by OSLOM_30-defined community as in (A). Edges are colored according to the OSLOM community to which the connecting region on the right belongs, with edge weight and transparency mapping to the average number of tracts between the two regions. (C) The structural connectome between the 15 overlapping regions is depicted, with line width and transparency indicating the log-transformed streamline count. (D) Each of the 15 overlapping nodes is highlighted in color on the brain’s surface, annotated with the same multitone circles as in (A) to indicate shared community membership.

As shown on the brain’s surface in Figure 4A, OSLOM_30 yielded an overlapping modular decomposition with seven modules, which we labeled as follows: (1) dorsomedial cortex, (2) limbic, (3) visual, (4) ventral attention and visual stream, (5) somatomotor, (6) insula, and (7) frontal pole. Of note, most individual regions within each right-hemisphere structural community are spatial neighbors, with modules thus forming spatially contiguous clusters. This is consistent with prior findings that spatially proximal regions are more likely to exhibit axonal connectivity between each other (Ercsey-Ravasz et al., 2013) and share similar connectivity patterns with the rest of the brain (Alexander-Bloch et al., 2019; Valk et al., 2020). The seven OSLOM_30-defined communities show substantial overlap with the seven canonical resting-state functional networks described in Yeo et al. (2011; see Supporting Information Figure S7 for overlap proportions shown as a heatmap), which is consistent with work from Chen, He, Rosa-Neto, Germann, and Evans (2008), showing that anatomical community structure, based on cortical thickness (not structural connectivity), is closely related to functional systems (Chen et al., 2008).

Relative to non-overlapping methods, a key benefit of OCDAs is their ability to identify overlapping nodes—that is, brain regions that bridge two or more different communities. Our OSLOM_30 decomposition identified 15 overlapping brain regions: *PreS*, *ProS*, *PeEc*, *PHA1*, *POS2*, *RSC*, *TE1a*, *PH*, *PGp*, *STSva*, *PFm*, *PGs*, *8Av*, *8BM*, and *9m*. These overlapping regions are highlighted in bold on the right-hemisphere cortical surface along network edges in Figure 4A, and the communities to which they were assigned are summarized in Table 3. Specifically, these overlapping regions were collectively assigned to six pairs of communities (e.g., “Frontal pole + limbic,” “Somatomotor + ventral attention and visual stream”), and one region was assigned to three communities (“Limbic + visual + dorsomedial”). The intra-hemispheric connectogram (with log-transformed streamline counts, cf. Preprocessing Structural Connectome Data section) in Figure 4B verifies that these overlapping regions exhibit structural connections across multiple modules. While the benchmarking analysis presented in Figure 3 (based on ENMI and sensitivity and specificity of assignment of overlapping status to nodes) does not directly support the accuracy of specific regions assigned to specific overlapping communities with “OSLOM,” the connectivity patterns in Figure 4B indicate that these regions are indeed structurally affiliated with regions that “OSLOM” assigned to the corresponding two (or three) modules. Examining intra-hemispheric structural connectivity specifically between these 15 overlapping nodes in Figure 4C reveals strong interconnectivity, consistent with the notion that overlapping regions integrate information across segregated brain networks—noting that these regions exhibit higher overall degree on average (cf. Supporting Information Figure S8A).

**Table 3.**
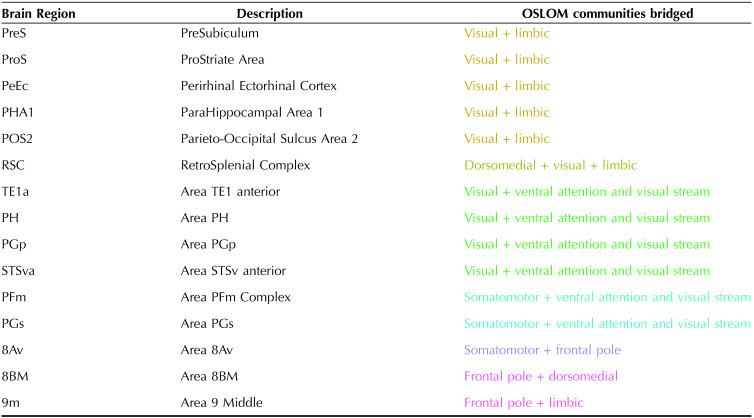
The 15 nodes that OSLOM_30 identified as overlapping across two or more structural communities in the right-hemisphere cortical connectome

The “+” sign denotes an overlap between the previous and subsequent listed communities.

Figure 4D plots the overlapping regions on the right-hemisphere cortical surface, revealing that the 15 nodes are evenly distributed along both anterior–posterior and medial–lateral axes. On average, each overlapping node comprised a larger surface area than a non-overlapping node (unpaired Wilcox rank-sum test, *P* = 0.03; cf. Supporting Information Figure S8B). Comparing the locations of these overlapping regions with those of the seven structural communities, we find that they sit at the spatial interface between adjacent communities. For example, the “Somatomotor + ventral attention” overlapping regions, *PFm* and *PGs*, sit at the ventral end of the “Somatomotor” module and the dorsal end of the “Ventral attention and visual stream” module. Together with the inter-module connectivity depicted in Figures 4A, 4B, this suggests that overlapping nodes can form a structural bridge for rapid integrative processing between the constituent brain networks—in which case, these regions could serve as cross-network “integrators” or “hubs” (Assem, Glasser, Van Essen, & Duncan, 2020; Puxeddu, Pope, Varley, Faskowitz, & Sporns, 2024; Sporns, 2013).

**Figure 5.**
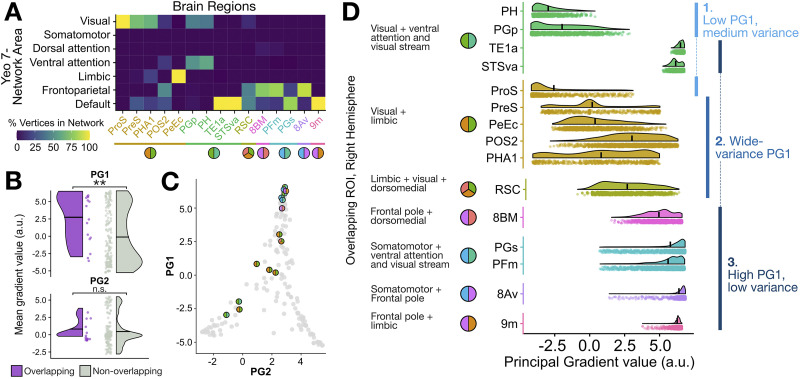
Regions participating in multiple structural communities generally sit higher in the human cortical hierarchy. (A) For the 15 cortical brain regions assigned to two or more modules, the percentage of vertices corresponding to each of the seven resting-state networks from Yeo et al. (2011) is shown as a heatmap. (B) The mean first (PG1) and second (PG2) principal gradient values computed across all vertices per region are shown in raincloud plots for overlapping regions (purple, 15 regions) and non-overlapping regions (gray, 164 regions). **, *P* < 0.01, Wilcox rank-sum test; n.s., *P* > 0.05. (C) The mean PG1 versus PG2 values are plotted per brain region, with overlapping regions indicated by multitone circles as in (A). (D) For each group of overlapping nodes (e.g., “Visual + cingulate and parahippocampal”), the PG1 values are plotted for all vertices as raincloud plots. a.u. = arbitrary units. In all plots, the black bar spanning each violin half represents the mean of the corresponding distribution. On the right-hand side of raincloud plots, nodes have been grouped into one of three categories based on the patterns of PG1 value distributions.

Based on the seminal work in Mesulam (1998)—and mounting evidence that “integrative hubs” contribute to multiple functional domains (Armas et al., 2024; Mecklenbrauck et al., 2024; Najafi et al., 2016; Zamora-López, Zhou, & Kurths, 2010)—we hypothesized that overlapping regions that OSLOM assigned to two or more structural communities would sit higher in the putative functional hierarchy of the cortex, such that their cross-community connectivity enables the exchange of information from distributed modules. To test this hypothesis, we first examined the extent to which each overlapping node mapped to each of the seven canonical resting-state functional networks from Yeo et al. (2011). As shown in Figure 5A, we found that roughly half of the overlapping nodes map primarily to the higher order default mode and frontoparietal (cognitive control) systems, which comprise association and paralimbic cortices. By contrast, the remaining overlapping regions correspond primarily to the visual network (a unimodal system) or the ventral attention and limbic networks (heteromodal systems). The differential network composition across the 15 overlapping nodes suggests a natural bipartition, with each half sitting toward either the lower or the higher end of the cortical functional hierarchy, respectively.

We next quantitatively compared the location of each overlapping node along the cortical hierarchy by examining the first and second PGs of functional connectivity (Margulies et al., 2016) provided in the *neuromaps* atlas (Markello et al., 2022; cf. Comparing Overlapping Nodes With Biologically Relevant Properties section). As described in Margulies et al. (2016), the first PG tracks a topographical hierarchy in the cortex, anchored by unimodal sensorimotor regions at the base and transmodal association regions at the top, with heteromodal regions supporting the domain-general processing along the middle; whereas the second PG distinguishes between primary modalities by separating auditory and sensorimotor from the visual regions. As shown in Figure 5B, overlapping brain regions identified by OSLOM_30 exhibited significantly higher PG1 values—averaged across all vertices per region—than non-overlapping counterparts (*P* = 0.004, Wilcox rank-sum test). This supports the notion that regions assigned to two or more intra-hemispheric structural communities sit higher in the topographical hierarchy of the cortex on average—with the caveat that external validation will be needed to confirm this, given the inherent limitations in the accuracy of specific community label assignments. Although there was no statistically significant difference in PG2 values between overlapping versus non-overlapping nodes (*P* = 0.14, Wilcox rank-sum test), plotting nodes in the PG1–PG2 space in Figure 5C revealed that overlapping regions sit within the visual–transmodal axis, with a notable lack of regions in the auditory region (characterized by low PG1 and high PG2 values). This supports the notion that regions bridging two or more structural communities generally sit higher in the functional hierarchy of the cortex.

The spread of PG1 values among overlapping regions is surprising, indicating that the nodes do not all sit narrowly at the top of the functional hierarchy of the cortex. Rather, the overlapping regions might be positioned across multiple hierarchical levels, which could allow them to act as intermediaries in integrating across systems that may sit at very different levels of the functional hierarchy. Indeed, Najafi et al. (2016) found that overlapping regions exhibit greater functional diversity than those participating in only one network. While the overlapping regions did not exhibit more variance in vertex-wise PG1 values compared with non-overlapping regions (as shown in Supporting Information Figure S9), we aimed to investigate the overall distribution of PG1 values across vertices in each overlapping region. As shown in Figure 5D, we found interesting and distinctive patterns of vertex-wise PG1 values in overlapping regions, which we separated into three distinct patterns: (a) low PG1 values with moderate variance, corresponding to *ProS*, *PH*, and *PGp*, which map primarily to the visual and ventral attention resting-state networks; (b) moderate PG1 values with high variance, corresponding to heteromodal integration of domain-general processing, in the tri-modular *RSC* node and in four of the bi-modular “Visual + limbic” nodes: *PreS*, *PeEc*, *POS2*, and *PHA1*; and (c) high PG1 values with low to moderate variance, corresponding to transmodal association of higher order functions, in the remaining nodes: *TE1a*, *STSva*, *8BM*, *PGs*, *PFm*, *8Av*, and *9m*. These distinctive patterns in the mean and variance of PG1 values suggest that overlapping regions generally sit higher in the putative functional connectivity hierarchy, potentially enabling more synergistic systems-level processing—although this pattern is not universal across all overlapping regions. Rather, our findings suggest that the role of a given overlapping node within its complex network topology may depend upon the two or more communities it bridges, along with other structural and functional properties, from cytoarchitecture to neurotransmitter receptor densities (Bazinet, Hansen, & Misic, 2023; Lawn et al., 2023).

### Comparing OSLOM With Louvain Demonstrates the Importance of Allowing Network-Bridging Overlaps

Having demonstrated that OSLOM_30 generates biologically plausible intra-hemispheric structural networks bridged by overlapping nodes, we next asked how much additional information this overlapping modular network decomposition provides beyond that of a conventional, non-overlapping partition. In particular, we wanted to understand whether the overlapping nodes assigned by OSLOM exhibit distinctive nodal metrics of network integration relative to those of a non-overlapping partition, such as participation coefficient, *P*, and within-module strength (*z*-score), *z* (Guimerà & Nunes Amaral, 2005). As originally described in Guimerà and Nunes Amaral (2005) and further extended to brain connectivity subsequently (Klahn et al., 2024; Sporns, Honey, & Kötter, 2007; van den Heuvel & Sporns, 2013b), comparing *P* and *z* can provide insight into the functional role that a given node may fulfill in its community, from peripheral hubs (low *z*, low *P*) through global connectors (high *z*, high *P*). Since the 15 OSLOM_30-defined overlapping nodes exhibited many connections across communities and between each other (cf. Figures 4B, 4C), we expected that they would sit in the “global connectors” (high *z*, high *P*) space.

**Figure 6.**
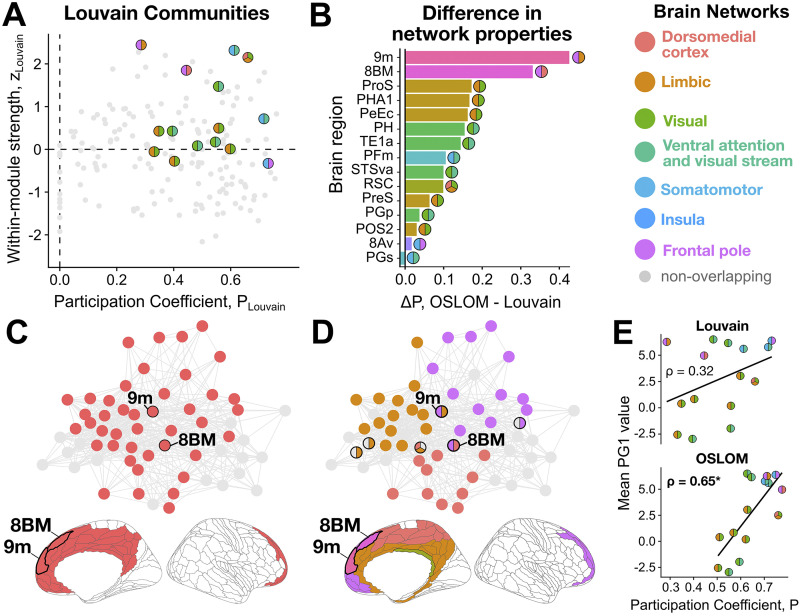
Overlapping nodes cannot straightforwardly be inferred from nodal metrics of a non-overlapping community decomposition. (A) Scatterplots for *P* versus *z* for every node in the right hemisphere cortical connectome using the Louvain (non-overlapping) partition. The overlapping nodes (obtained by OSLOM_30) are marked in two- or three-tone circles, with the two (or three) colors indicating the pair of communities bridged by the node in the OSLOM decomposition. (B) The difference in *P*_OSLOM_ versus *P*_Louvain_ is compared for each OSLOM-identified overlapping region, ranked by the value of Δ*P*. (C) Louvain assigns nodes *8BM* and *9m* to a community of 46 total nodes spanning frontal, cingulate, and dorsomedial cortices (n.b., only a subset of these nodes are shown that are structurally connected to *8BM* and/or *9m*), whereas (D) OSLOM subdivides this set of nodes into three communities, collectively bridged by *8BM* and *9m*. The two-tone circles for *8BM* and *9m* indicate the two structural communities to which they were assigned. Circles with pink-gray or orange-gray coloring indicate an overlap between the 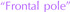 or 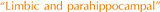 and other modules, respectively. Completely gray circles represent non-overlapping nodes. Nodes *8BM* and *9m* are annotated with bolded edges on the brain’s surface below the network plots in (C) and (D). (E) For each overlapping node, its mean PG1 value is compared with its participation coefficient from either Louvain or OSLOM partitioning. Spearman’s *ρ* is shown for each comparison; *, *P* < 0.05.

For comparison with the OSLOM_30 overlapping partition, we selected the Louvain clustering method (Blondel et al., 2008) for consistency with the benchmarking analysis. The Louvain method yielded a non-overlapping modular decomposition, from which *z* and *P* were derived for each node (as described in the Comparing Overlapping and Non-Overlapping Modular Partitions section). Using a representative decomposition with the resolution parameter *γ* = 1—which is the default in the Brain Connectivity Toolbox (Rubinov & Sporns, 2010) and is supported by our robustness analyses in Supporting Information Figure S4—we identified six non-overlapping Louvain communities in the right hemisphere, shown on the cortical surface in Supporting Information Figures S4B, S4C. We also performed a robustness analysis with *γ* = 1.5 to enforce a seven-module (non-overlapping) decomposition in the right hemisphere, and the results were comparable with those obtained with *γ* = 1 (cf. Supporting Information Figures S4D–S4G), so we focus on the *γ* = 1 Louvain results hereafter. To characterize nodes according to Louvain versus OSLOM partitioning, we compared *P* versus *z* scores for each node. We show each node in the *P*_Louvain_ − *z*_Louvain_ space in Figure 6B, where overlapping nodes (colored circles) are compared with non-overlapping nodes (gray circles). Most of the OSLOM_30-defined overlapping nodes exhibit *z*_Louvain_ > 0 (11 out of 15 nodes), indicating strong within-Louvain module connectivity relative to other regions. However, only half of the overlapping nodes (eight out of 15) exhibit *P*_Louvain_ > 0.5, and even those do not stand out as exhibiting particularly high *P*_Louvain_ relative to non-overlapping nodes. For example, regions *8BM* and *9m*—judged as overlapping by OSLOM_30—exhibit strong within-Louvain module connectivity (high *z*_Louvain_) yet sit toward the lower end of the *P*_Louvain_ distribution. This is surprising, as we expected regions that OSLOM_30 identified as overlapping to show a greater spread of connectivity to nodes across multiple Louvain-defined modules (i.e., closer to “global connectors” or at least “provincial hubs”).

Of note, *P* is a label-invariant measure, as it focuses on the relative frequency of connected neighbor nodes in each module rather than the identity the modules themselves—meaning that it is still valid regardless of whether the corresponding community detection algorithm (OSLOM or Louvain, in this case) recovers the ground-truth community structure from the empirical right-hemisphere structural connectome. To compare network properties with the OSLOM partition, we adapted the participation coefficient, *P*, to allow each overlapping node to be counted as part of its constituent two or three communities (noting that the specific module assignment label does not affect the value of *P*). With this adaptation (cf. Comparing Overlapping and Non-Overlapping Modular Partitions section), *P* will increase by definition, so we focus on the relative differences in the magnitude of change (i.e., Δ*P*) in Figure 6B. Regions *9m* and *8BM* stood out as the two regions with the greatest increase in *P* as measured by OSLOM relative to Louvain—prompting more detailed investigation of these regions. To investigate the structural changes in modular structure that may have underpinned these substantial increases in participation coefficient for *9m* and *8BM*, we further examined their “sub-network graphs”—composed of the cortical regions sharing intra-hemispheric structural connections to one or both of *8BM* and *9m*. These subnetworks that include *9m* and *8BM* are plotted in Figures 6C and 6D, for the Louvain and OSLOM modular decompositions, respectively. As shown in Figure 6C for the Louvain decomposition, both *9m* and *8BM* were assigned to the same community, composed, in total, of 46 right-hemisphere nodes (of which 40 are included in this subnetwork) that collectively span frontal, cingulate, and dorsomedial cortices. We find that the same nodes depicted in the OSLOM decomposition in Figure 6C are partitioned into two distinct communities that are bridged by *9m* (the “Frontal pole” and “Limbic” modules) and another two by *8BM* (the “Frontal pole” and “dorsomedial cortex”).

These distinct modular decompositions demonstrate that OSLOM can disentangle multiple partially overlapping modules, capturing more complex yet subtle structure—which can be lost with a non-overlapping algorithm that cannot accommodate overlaps and therefore merges nodes into one larger module. While OSLOM detects different structures in the network and, thus, yields different network nodal statistics like the participation coefficient, we next aimed to investigate whether the participation coefficient derived from Louvain or OSLOM may better capture other independently measured properties of brain organization among overlapping nodes, such as the position along the cortical functional hierarchy (Margulies et al., 2016; Mesulam, 1998). As plotted in Figure 6E for the 15 overlapping right-hemisphere nodes identified by OSLOM, the mean PG1 values (cf. Figures 5B–5D) were significantly correlated with *P*_OSLOM_ (Spearman’s *ρ* = 0.65, *P* = 0.01) but not with *P*_Louvain_ (*ρ* = 0.32, *P* = 0.24). Similarly, we found no association between PG1 values and *P*_Louvain_ in our robustness analysis with *γ* = 1.5 (*ρ* = 0.37, *P* = 0.2; cf. Supporting Information Figure S4G). This indicates that the overlapping regions identified by OSLOM exhibit a greater degree of cross-module (intra-hemispheric) connectivity, which, in turn, positively correlates with position in the putative functional hierarchy of the cortex. The same relationship holds when examining all 180 regions from the HCP-MMP1 right-hemisphere atlas (Glasser et al., 2016): As shown in Supporting Information Figure S10, all mean PG1 values were significantly correlated with *P*_OSLOM_ (Spearman’s *ρ* = 0.33, *P* = 6 × 10^−6^) but not with *P*_Louvain_ (*ρ* = 0.01, *P* = 0.9). In other words, testing the hypothesis that the participation coefficient increases along the cortical functional hierarchy yielded different conclusions using an overlapping versus non-overlapping decomposition. Only the OSLOM-derived participation coefficient *P* provides evidence for this hypothesis, with regions broadly increasing in their *P* from the unimodal end of the hierarchy up to the transmodal, associative end. Collectively, our comparison of metrics derived from OSLOM versus Louvain partitions underscores the utility of flexible overlapping decompositions for interpreting the resulting structural networks and derived nodal statistics, which are widely interpreted across the network neuroscience literature.

## DISCUSSION

This work introduces a general data-driven framework for selecting an OCDA for a given application. We further demonstrate how such overlapping modular decompositions can provide novel insights into the structure of human (intra-hemispheric) cortical connectivity. For a given target empirical dataset—here, the right-hemisphere structural connectome—the proposed method systematically compares how well each OCDA recapitulates the ground-truth overlapping community structure across benchmark networks generated from properties of the target network. This problem of calibrating methods to data relates to the concept of an algorithmic “footprint” characterized in related work on other data types (Smith-Miles, Baatar, Wreford, & Lewis, 2014), which captures the differing regions (or “footprints”) in the data space in which different types of algorithms exhibit strong performance. Acknowledging that each OCDA makes different assumptions about network structure and inter-regional communication—and, therefore, that no OCDA can exhibit good performance on every type of network (cf. the “no free lunch” theorem; McCarthy et al., 2020; Wolpert & Macready, 1997)—our method circumvents the typical subjectivity in selecting an OCDA for a given problem, at the expense of constraints imposed on the simulated network ensemble by the benchmark generation process (Lancichinetti & Fortunato, 2009). This demonstrates the importance of empirically tailoring OCDAs to a given network problem at hand, rather than relying on overly general statements about the relative performance of one algorithm over another (Peel et al., 2017). We demonstrate an application of our approach to the structural connectome of the human right-hemisphere cortex, where we select an appropriate OCDA algorithm (OSLOM) from an ensemble of brain-like networks. We then show how OSLOM quantifies new types of structure in brain networks, particularly by identifying overlapping regions that bridge across multiple biologically sensible modules and sit higher in the topographical cortical hierarchy. Furthermore, the OSLOM decomposition yields nodal metrics that better distinguish these overlapping regions and align with independent measures of cortical organization, supporting the hypothesized hierarchical variation of nodal participation.

Using OSLOM to infer the overlapping community structure of the right-hemisphere cortical connectome, we obtained a useful and interpretable representation of the network structure with seven biologically plausible processing modules, collectively bridged by 15 integrative overlapping nodes. While our method cannot directly confirm the accuracy of these identified overlapping nodes in the absence of a ground-truth empirical right-hemisphere community structure, these findings are generally in line with previous studies mapping structural and functional connectomic data. For example, prior work suggests that higher order integrative regions like *PFm* and *PFs*—which were identified by OSLOM as exhibiting community overlap between the “somatomotor” and “ventral attention and visual stream” networks—serve as important functional and cytoarchitectonic transition zones between neighboring brain areas (Igelström & Graziano, 2017; Wig, Laumann, & Petersen, 2014). In addition to *PFm* and *PFs*, Regions *8BM* and *9m* in the dorsomedial prefrontal cortex also exhibited among the highest PG of functional connectivity (PG1) values (Margulies et al., 2016) of the regions we evaluated (cf. Figure 5C). For these nodes, the intra-hemispheric structural connectivity overlaps may provide physical substrates for transmodal and/or associative functional integration. Region *8BM*, which showed overlapping community affiliation with the “frontal pole” and “dorsomedial cortex” modules here and is a part of the frontoparietal resting-state network (Yeo et al., 2011), has previously been highlighted as a higher order cognitive control hub (Luo et al., 2024). Moreover, Assem et al. (2020) showed that *8BM*, *PFm*, and *PGs* are also components of the “multiple-demand” network that orchestrates a variety of cognitive tasks, underlying flexible organization and cognitive control. Our findings are therefore consistent with the hypothesis that overlapping regions span multiple structural communities to enable functional integration.

However, some overlapping nodes’ PG1 values positioned them closer to heteromodal limbic regions (e.g., *POS2* and *RSC*) or even lower order unimodal sensorimotor regions (e.g., *PH* and *PGp*). Despite sitting lower in the first putative topographical hierarchy of the cortex than other overlapping nodes, *POS2* (a component of the parieto-occipital sulcus in the HCP-MMP1 atlas; Glasser et al., 2016) participates in individual-specific functional network overlaps and is distinct from neighboring regions based on its cortical microstructure, functional connectivity, and task activation (Bijsterbosch et al., 2023). *RSC* (standing for “retrosplenial cortex” in the HCP-MMP1 atlas; Glasser et al., 2016) is noteworthy as the only region identified to bridge across *three* structural networks here: “limbic,” “visual,” and “dorsomedial cortex.” The RSC exhibits dense inter-network connectivity (Alexander, Place, Starrett, Chrastil, & Nitz, 2023) and plays a critical integrative role in synthesizing viewpoints to process one’s environment (Chrastil, 2018; Park & Chun, 2009). Together with the literature, our findings suggest that *POS2* and *RSC* serve as integrative transmodal hubs that bridge two or three distinct networks, respectively. For the lower PG1 regions—*PH* in the temporo-occipital junction and *PGp* in the inferior parietal cortex—the potential role for structural overlap is less clear from the literature. *PGp* is a part of the angular gyrus (AG) believed to mediate semantic processing (Jefferies, 2013; Kuhnke et al., 2023), although evidence suggests thar it plays less of an “integrative” role than other AG subregions (Niu & Palomero-Gallagher, 2023). Region *PH* contributes to visual processing from the foveal field (Benson et al., 2018), in line with the shared connectivity between the “visual” and “ventral attention and visual stream” networks identified herein.

Together, the differing patterns in functional connectivity PG1 values among the overlapping nodes suggest a wide spectrum of functions served by cross-network structural bridges, which likely depend on a variety of factors—including dynamic brain state, cytoarchitecture, receptor densities, and functional properties of the underlying brain systems. In support of this, Najafi et al. (2016) described different “bridging” behaviors exhibited across different overlapping regions based on the functional connectome, and Bijsterbosch et al. (2023) found evidence for both inter-community coupling as well as “interdigitation” (i.e., spatial interweaving of two networks) among areas of overlap between functional modules. Previous work has also suggested that such regions play a key role in information integration across brain modules and dynamical network states (Bullmore & Sporns, 2012; de Reus, Saenger, Kahn, & van den Heuvel, 2014; Shine et al., 2016; van den Heuvel & Sporns, 2011, 2013a). Given this heterogeneity, future study is warranted to more fully characterize how the nature of overlapping regions relates to their own intrinsic structural, functional, and/or cytoarchitectonic properties, as well as those of the network communities they span.

The relatively low number of overlapping regions (15/180) is notable, with interesting implications in terms of both methodology and biology that warrant future investigation. While OCDAs have been applied to structural connectomes in mice (Sotiras et al., 2015) and macaques (Armas et al., 2024), to our knowledge, this is the first study to apply an OCDA to the human structural connectome derived from diffusion MRI. In light of this, there is limited precedent for directly comparing our observed overlap ratio with previous work. However, we speculate that the variance-based consensus thresholding method applied here—which was necessary to tune the required parameters to a single empirical adjacency matrix for the benchmark generation algorithm from Lancichinetti and Fortunato (2009)—might contribute to the low proportion of overlapping nodes in the right cortical structural connectome, as sparsification reduces the opportunity for nodes to participate in multiple communities. Indeed, Gu et al. (2022) applied an OCDA to individual resting-state (functional) connectomes, reporting much higher overlap densities across individuals (80–100 out of 246 nodes) with age-related differences. By contrast, Li et al. (2016) implemented group-averaging and reported only 16 overlapping nodes out of the 90 regions in the automated anatomical atlas (AAL) parcellation (Tzourio-Mazoyer et al., 2002). These differences in overlapping region density are likely attributable to factors including algorithm choice, imaging modality, and whether the analyses are performed on individual or group-level networks. Biologically, the minimal overlap in our results suggests intriguing questions for future work about the efficiency and specificity of structural brain organization. Given the high metabolic cost of maintaining long-range axonal connections (Bullmore & Sporns, 2012), it may be that structural modules are relatively segregated, with only a minimal set of overlapping “bridge” regions facilitating inter-modular communication. Of note, OSLOM identified at least one overlapping node per structural community (with the exception of the insular network), suggesting that while rare, overlaps are distributed across modules.

Comparing intra- and inter-module network statistics, like *P* and within-module *z*, highlighted distinct network properties derived from an overlapping versus non-overlapping modular partition. Our results indicate that the inflexibility of the Louvain decomposition—which seeks the optimal non-overlapping partition—can “force” an overlapping modular structure into a single larger community in attempting to represent an underlying overlapping connectivity structure within the constraints of a non-overlapping partition, consistent with prior work with fMRI (Najafi et al., 2016). In one example of this phenomenon (Figure 6C), the Louvain partition consolidated regions *9m* and *8BM* into a singular 46-node community, with high *z* and low *P* (i.e., high intra-modular connectivity but low inter-modular participation). We also note that enforcing a seven-module Louvain decomposition with *γ* = 1.5 still placed *9m* and *8BM* in a singular, 32-node community. More generally, the fact that all the OSLOM-defined overlapping nodes were not distinctively concentrated in a specific area in the *z*_Louvain_ − *P*_Louvain_ space underscores how non-overlapping community detection methods can yield suboptimal representations of the integrative, cross-module connectivity within a network structure. In other words, the properties of a hard partition (like the within-module degree or participation coefficient in a non-overlapping decomposition) are insufficient to identify overlaps between communities. Since *P* is agnostic to the specific labels ascribed to each discovered community, results obtained with this measure do not hinge on the accuracy of community assignment by a given community detection algorithm, lending further credence to the performance of OSLOM for the right-hemisphere structural connectome. We note that *z* and *P* were both developed for non-overlapping community structure—thus requiring us to make slight modifications to these algorithms to account for overlapping nodes. Future work developing network characteristics that are readily amenable to both overlapping and non-overlapping algorithms would enable more rigorous direct comparison between two resulting decompositions.

Our methodology and results come with assumptions and limitations that should be interpreted in the context of this work. First, we consider limitations in our simulated network benchmark analysis and proposed future directions to address them. We assume that the generated ensemble of benchmark networks reflects the overlapping community structure of the target empirical network and, in turn, that the top-performing OCDA on the benchmark ensemble will also perform well on the target network—such that ensemble properties are crucial to determining the “best” OCDA. Moreover, we used the generative network model of Lancichinetti and Fortunato (2009), which forces a power-law degree distribution that may not be optimally suited for many real-world networks—including the human connectome studied here, in line with previous findings (Gajwani et al., 2023; Gastner & Ódor, 2016; Hagmann et al., 2008). The model also does not incorporate other types of network properties, such as hierarchical community structure, which is a reported feature of brain network organization (Meunier, Lambiotte, & Bullmore, 2010). In addition, given a generative model of overlapping community structure in networks, we had to make subjective choices about how to sample from the corresponding distribution over networks numerically. Here, we sampled from sensible ranges of the relevant parameters—including community size distribution *τ*_2_, mixing parameters *μ*_*t*_ and *μ*_*w*_, and the number of overlapping nodes *O*_*n*_—although these remain choices that can affect the assessment of the resulting OCDAs and could be explored more thoroughly in future work. As more sophisticated generative network models are developed that can incorporate a larger range of topological constraints, they can readily be incorporated into our framework to allow more precise calibration of OCDAs to target empirical data.

Second, we consider ways in which future work could extend the application of OCDAs introduced here beyond the scope of the present study. Future work should investigate the consistency of community detection assignments of a given OCDA and also between different algorithms (including for different parameter configurations). Similarly, we considered a hand-selected range of parameter values for each algorithm, although future extensions of this work could fit parameters for each OCDA using a training ensemble of benchmark networks. Such parameter tuning is likely to improve the ENMI values in general, allowing for more precise calibration of high-performing parameter values for each OCDA to a given application. More broadly, future work could expand beyond the five broad OCDA classes examined here to incorporate alternative methods that warrant investigation (Gopalan & Blei, 2013; Ma, Yang, Zhou, Zhang, & Zhang, 2021; Yuan, Wang, & Song, 2016).

Finally, we consider limitations related to our empirical structural intra-hemispheric connectome analysis, which generally reflect issues inherent to structural connectomes estimated from diffusion MRI. Tractography-based connectivity estimates can be biased by systematic anatomical differences between cortical and subcortical structures, including the small size of nuclei relative to diffusion imaging voxels and different properties of laminar organization and the gray–white matter interface (Rosen & Halgren, 2021; Sotiropoulos & Zalesky, 2019), motivating our focus on cortical intra-hemispheric connectivity. Limitations in reconstructing long-range fibers also guided our decision to focus on intra-hemispheric connections within the right hemisphere (noting highly similar connectomic network properties between the two hemispheres, such that this choice was arbitrary and unlikely to affect results). Indeed, it is relatively common practice in structural connectomics to analyze each hemisphere separately—or focus on a single hemisphere altogether—to improve anatomical reliability (Arnatkeviciute et al., 2021; Betzel et al., 2013; Deco & Kringelbach, 2020; Pang et al., 2023; Vohryzek et al., 2025). However, this precluded our ability to examine homotopic and other inter-hemispheric connections, both of which are core components of brain organization (Hagmann et al., 2008) and contribute to dysfunction in diverse pathologies (Jiang et al., 2019; E. Wang et al., 2025; Zhang et al., 2015). Therefore, extending these analyses to networks that incorporate inter-hemispheric connections in the cortex, subcortex, and cerebellum represents an important direction for future work to characterize the distributed modular architecture on the scale of the whole brain (Dimitriadis, Messaritaki, & Jones, 2021; Sanchez-Rodriguez et al., 2021).

Indeed, the seven distinct modules identified by OSLOM in the present study are generally spatially contiguous, with the exception of the “visual” and “ventral attention and visual stream” networks. Moreover, all of the overlapping nodes sit at the spatial interface between their constituent and adjacent structural communities, suggesting they form connectivity “bridges.” This finding is consistent with similar findings reported in the macaque structural connectome (Armas et al., 2024); however, future work should investigate whether this spatial contiguity in overlapping nodes arises from general biological patterns of brain organization and/or from the inherent contiguity of the specific parcellation atlas analyzed. For example, we applied the multimodal parcellation from Glasser et al. (2016), motivated by its strong neurobiological validity in integrating properties of local microstructure and global functional activity to delineate contiguous cortical regions. However, the use of any specific parcellation imposes spatial and topological constraints on network analyses; in particular, Bijsterbosch et al. (2020) highlighted how the choice of parcellation scheme can influence resulting patterns of (functional) connectivity and network topology.

On a related note, we found that the average overlapping node occupied significantly more cortical surface area than the average non-overlapping node. A larger surface area can lead to an increased number of streamlines assigned to that region in the tractography estimation process (Aerts et al., 2018; Gajwani et al., 2023), and the overlapping regions do, indeed, have a higher degree on average. However, both region size and parcellation atlas can affect nodal statistics and the spatial location of overlapping regions within the brain (Collin, Kahn, de Reus, Cahn, & van den Heuvel, 2014; Messé, 2020). To more precisely resolve these biological and methodological questions, future work should extend our analyses using alternate parcellation schemes—including those defined specifically based on structural connectivity (Allen, Zhang, & Nobel, 2024) or the intrinsic geometry of the cortex (Pang et al., 2025)—to assess the robustness of spatial patterns in overlapping community structure.

The method introduced here can be used to tailor overlapping community detection methods to network data, facilitating the additional insights that OCDAs can provide to diverse application areas. In our application to the human cortical right-hemisphere connectome, our results highlight the importance of uncovering meaningful overlapping network partitions that better capture how the brain balances functional integration and segregation on a static backbone of structural connectivity. Future work could map out such a space for empirical networks with overlapping modular structure (Agarwal, Villar, & Jones, 2010; Avena-Koenigsberger, Goñi, Solé, & Sporns, 2015) to more comprehensively characterize which parts of network space are best suited to which OCDAs. With this work, we also provide a comprehensive and extendable code base for overlapping community detection, which we hope will be expanded upon in future work, incorporating improvements in both new types of OCDAs (including those that incorporate higher order interactions; Puxeddu et al., 2024) and in overlapping benchmark network-generating algorithms.

## ACKNOWLEDGMENTS

The authors thank Mac Shine, Isabella Orlando, Joshua Tan, and Christopher Whyte for their valuable input on data visualization and biological interpretation.

## SUPPORTING INFORMATION

Supporting information for this article is available at https://doi.org/10.1162/NETN.a.39.

## AUTHOR CONTRIBUTIONS

Annie Gilmore Bryant: Formal analysis; Validation; Visualization; Writing – original draft. Aditi Jha: Formal analysis; Methodology; Validation; Visualization; Writing – original draft. Sumeet Agarwal: Conceptualization; Formal analysis; Supervision; Validation; Writing – review & editing. Patrick Cahill: Formal analysis; Visualization; Writing – review & editing. Brandon Lam: Formal analysis; Writing – review & editing. Stuart Oldham: Data curation; Formal analysis; Visualization; Writing – review & editing. Aurina Arnatkevičiūtė: Data curation; Formal analysis; Writing – review & editing. Alex Fornito: Conceptualization; Supervision; Writing – review & editing. Ben Fulcher: Conceptualization; Supervision; Writing – original draft.

## FUNDING INFORMATION

Annie Gilmore Bryant, Australian Government , Award ID: Research Training Program Stipend (International) (SC3229).

## Note

^1^ https://github.com/DynamicsAndNeuralSystems/OverlappingCommunityDetection_HCP.

## Supplementary Material



## TECHNICAL TERMS

Hub node: A node with many connections across communities, playing a central role in linking different parts of the network.
Community detection algorithm: A computational method to identify groups of nodes (e.g., brain regions) that are more strongly connected to each other than to the rest of the network.
Transmodal: A region or network that receives and integrates input from multiple functional networks.
Modular architecture: A network structure in which nodes are organized into distinct groups with dense internal connectivity, which serve different specialized functions.
Integration: The coordinated activity or information flow across different brain regions or network modules.
Segregation: The separation of anatomical structures and/or functional processes into distinct brain areas with limited interaction.
Ensemble of synthetic benchmark networks: A collection of simulated networks designed to emulate properties of the original network to compare algorithm performance.
Stochastic algorithm: A method that includes random processes, meaning that the outcome can vary with the same input data based on initialization conditions.
