# Supplementary material for: Benchmarking overlapping community detection methods for applications in human connectomics
Source: Netw Neurosci. 2026 Jan 8;10(1):25–61. doi: 10.1162/NETN.a.39 (PMC12798651; doi:10.1162/NETN.a.39)
Supplement: Supplementary file 1 [file netn-10-1-25-s001.pdf]

Supplementary figures for 'Benchmarking overlapping community detection methods for applications in human connectomics'

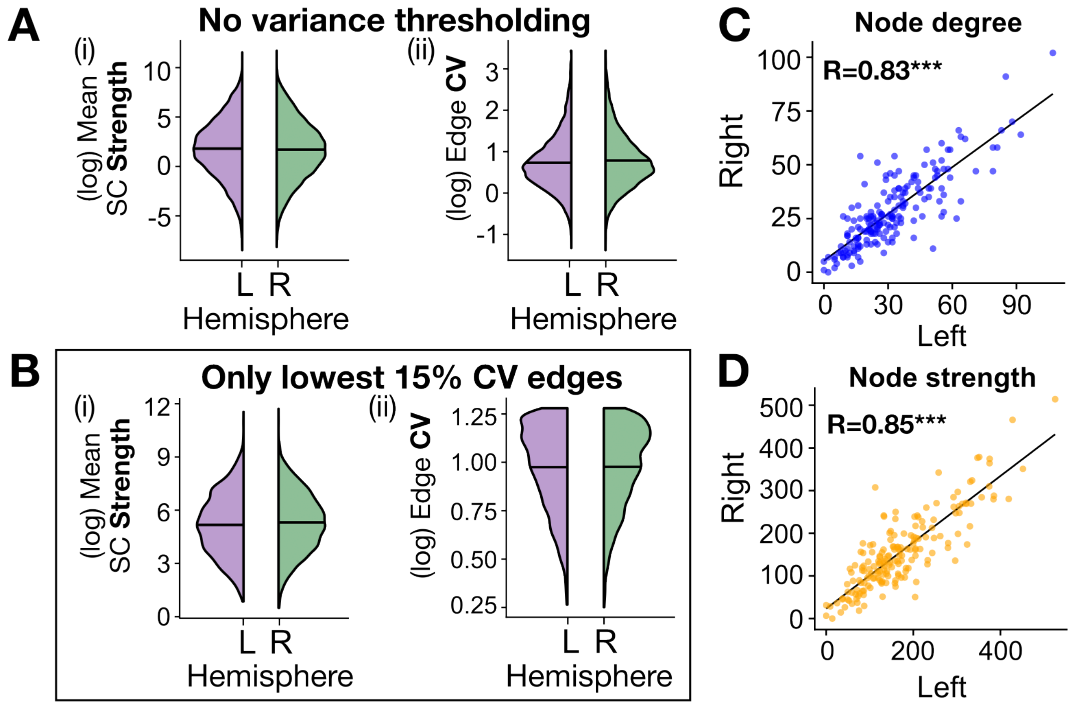

Figure S1: The left and right cerebral hemispheres exhibit highly similar intra-hemispheric structural connectivity profiles. A. For the left (purple) and right (green) hemispheres, the distribution of (i) log-transformed mean edge strengths, averaged across the group of N=973 participants, and (ii) the edge-wise CV values without any thresholding. **B.** As in **A**, after filtering to edges ranking in the lowest 15% of CV values (as in the main text).

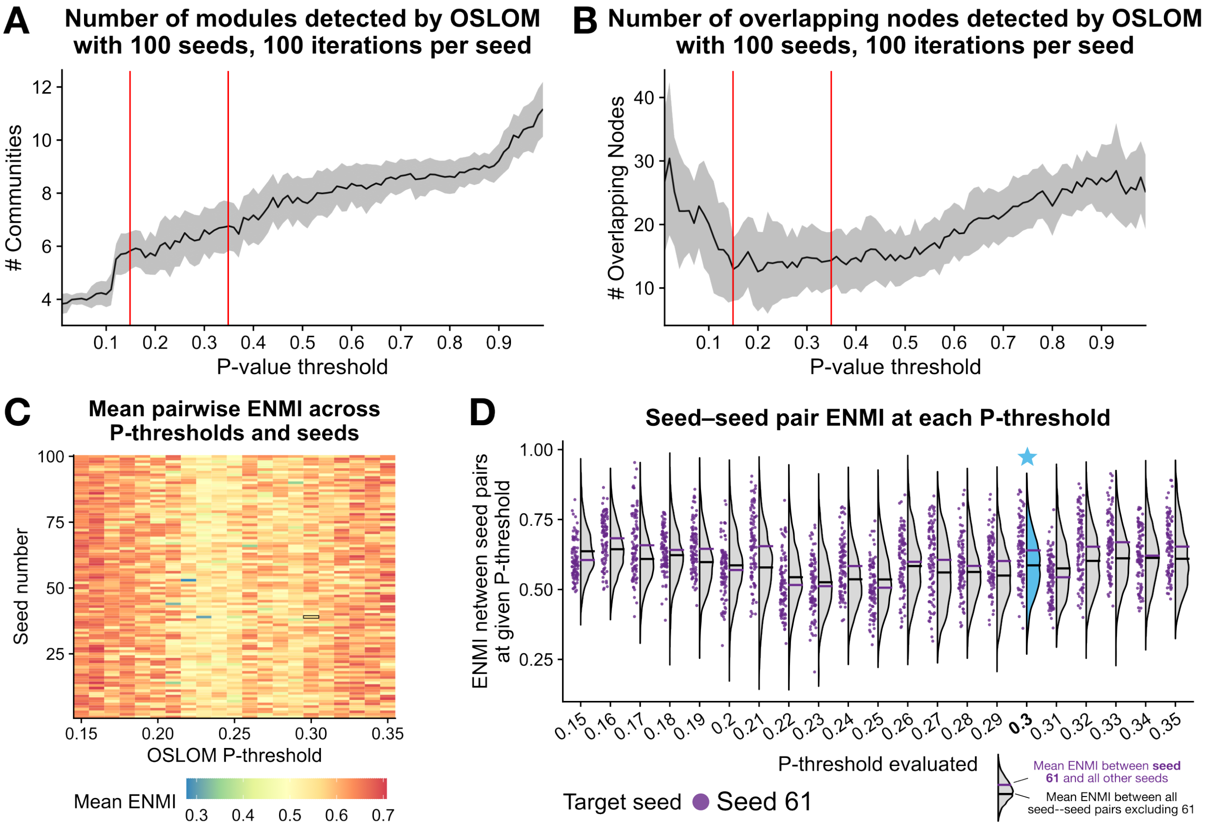

Figure S2: Parameter sweeps support the selection of $P = 0.3$ and seed number 61 for OSLOM. **A.** For each P-value threshold from 0.01 to 1, the mean number of communities that OSLOM identified from the empirical human cortical connectome is shown as a line plot. The shaded gray ribbon indices $\pm$ 1 SD and the red lines highlight the $0.15 \leq P \leq 0.35$ range. **B.** For each P-value threshold from 0.01 to 1, the mean number of nodes that OSLOM identified as overlapping across two or more communities is shown as a line plot. The shaded gray ribbon indices $\pm$ 1 SD and the red lines highlight the $0.15 \leq P \leq 0.35$ range. **C.** For the $0.15 \leq P \leq 0.35$ range, the mean pairwise ENMI between each seed and all 99 other evaluated seeds is plotted as a heatmap. The color range indicates that the mean ENMI values range between $[0.27, 0.71]$, with an average of 0.58 ± 0.06 (arbitrary units). **D.** For the final seed selection (61), the pairwise ENMI between seed 61 and each of the 99 other seeds is depicted as a raincloud plot. Specifically, for each P-threshold from $0.15 \leq P \leq 0.35$, there are 99 purple points corresponding to the ENMI between seed 61 and each of the 99 other seeds. The gray violins correspond to the ENMI distributions across all 4950 pairs of seeds from 1 to 100 (excluding self–pairs). Within each violin, the black bar measures the mean ENMI between all seed–seed pairs excluding seed 61, while the purple bar indicates the mean ENMI between seed 61 and all other seeds. The violin at $P = 0.3$ is highlighted in blue with a star as this is the final P-threshold choice in combination with seed 61, which yielded a mean ENMI of 0.64 ± 0.11, compared to the average across all other seeds of 0.59 ± 0.10.

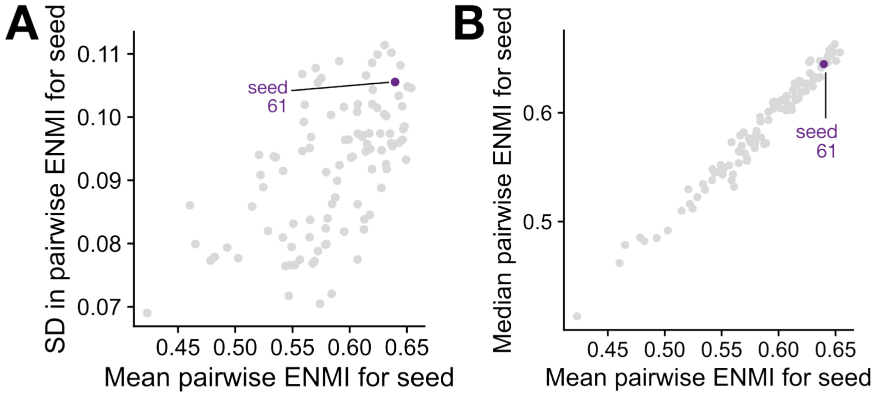

Figure S3: ENMI exhibits a heteroskedastic relationship for OSLOM applied to the right-hemisphere structural connectome, but the mean and median are very closely related. A. The mean (x-axis) and standard deviation (y-axis) of ENMI values for each of 100 initialization seeds supplied to OSLOM with the empirical right-hemisphere cortical connectome. **B.** The mean (x-axis) and median (y-axis) of ENMI values, as in **A**.

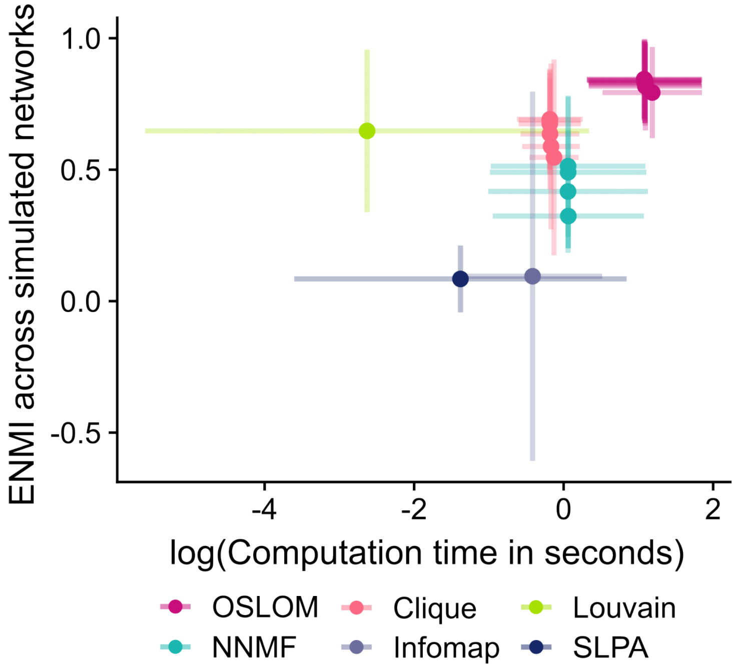

Figure S5: OSLOM with different thresholds consistently require the longest time to run, but yield the highest overall ENMI in the benchmark network ensemble. The mean computation time (in log(10)-transformed number of seconds) is plotted on the x-axis against the mean ENMI on the y-axis, each averaged across the 1,000 simulated networks in the benchmark ensemble. The cross-bars indicate ± 1 standard deviation for computation time (x-axis) or ENMI (y-axis).

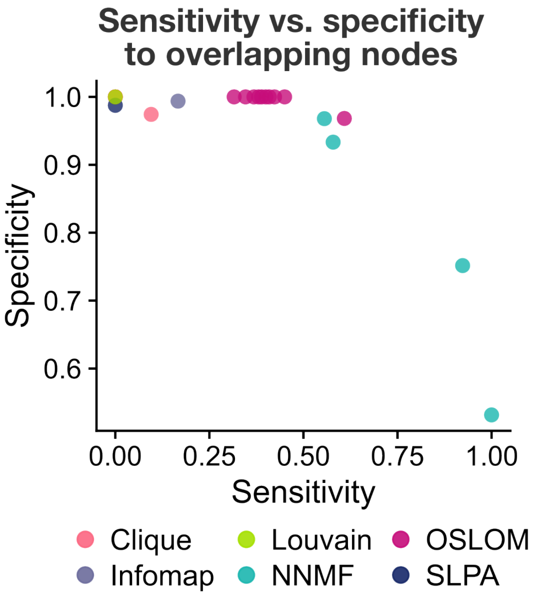

Figure S6: OSLOM yields a good balance between sensitivity and specificity in identifying ground-truth overlapping nodes across the benchmark ensemble. The mean sensitivity is plotted on the x-axis against the specificity on the y-axis, each averaged across the 1,000 simulated networks in the benchmark ensemble.

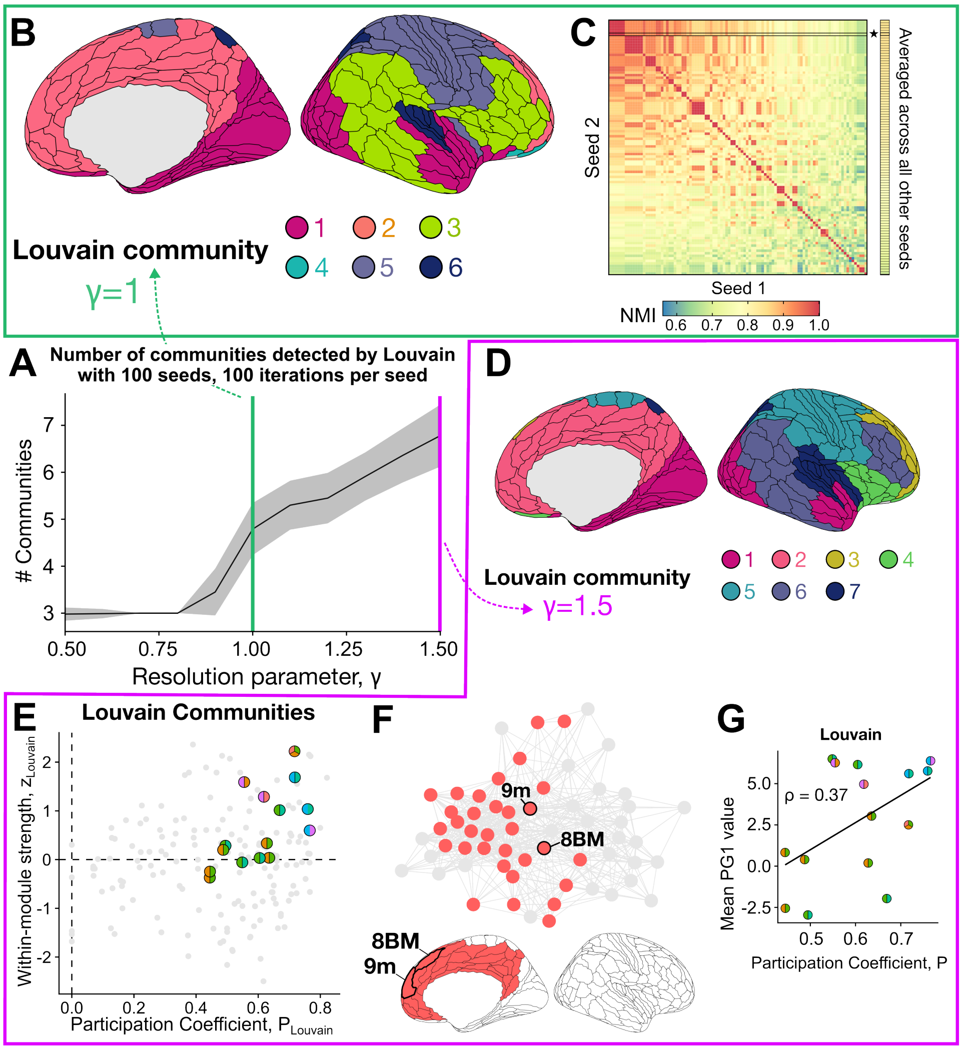

Figure S4: Louvain partitioning with $\gamma = 1$ yields a representative six-community decomposition, with comparable properties to that of a seven-community decomposition with $\gamma = 1.5$. A. For the resolution parameters $\gamma = [0.5, 0.6, \ldots 1.5]$, Louvain partitioning was performed using 100 initialization seeds each. The mean number of detected communities per γ value is plotted as a black line, with the shaded ribbon indicating ± 1 standard deviation. The green line at $\gamma = 1$ is included to guide visual interpretation for this threshold selection parameter, with a representative six-community decomposition projected onto the cortical surface in **B**. **C.** The result in **B** corresponds to the seed number 98, which was selected based on its maximal normalized mutual information (NMI) value to all other evaluated seeds, as shown in the heatmap. The pairwise NMI values are depicted as a heatmap, with the selected seed (98) outlined in bold with a star. **D.** To compare our seven-community OSLOM decomposition with a seven-community Louvain decomposition specifically, we also evaluated a maximally representative seed (seed=91) with the threshold $\gamma = 1.5$. These seven communities are projected onto the right cortical surface here. **E.** Scatter plots for P_{Louvain} versus z_{Louvain} for every node in the right hemisphere cortical connectome, using $\gamma = 1.5$. The overlapping nodes (obtained by `OSLOM-30`) are marked in two-tone circles, with the two colors indicating the pair of communities bridged by the node. **F.** Louvain (with $\gamma = 1.5$) assigns nodes *8BM* and *9m* to a community of 32 total nodes spanning frontal, cingulate, and retrosplenial/dorsomedial cortices (n.b., only a subset of these nodes are shown that are structurally connected to *8BM* and/or *9m*). Gray circles in the network plot represent nodes that are structurally connected to *8BM* and *9m* but not assigned to the same Louvain module. **G.** For each overlapping node identified by `OSLOM-30`, its mean PG1 value is compared with its P_{Louvain}. Spearman's $\rho = 0.37, P = 0.17$.

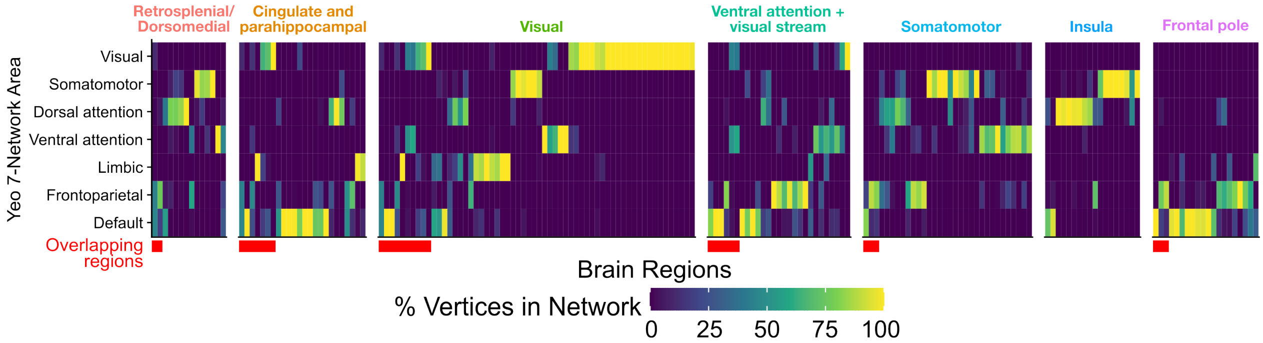

Figure S7: For each brain region, the proportion of vertices mapping to each of the 7-Network areas from Yeo et al. [116] was computed, with the percentages indicated as heatmap tile values. Brain regions are grouped along the x-axis by OSLOM-identified structural module, with the 'overlapping' regions to the left of each cluster (annotated by the red bar below the heatmap). Note that overlapping regions were included as columns in each of the corresponding OSLOM modules, such that each overlapping region appears two or three times in the heatmap.

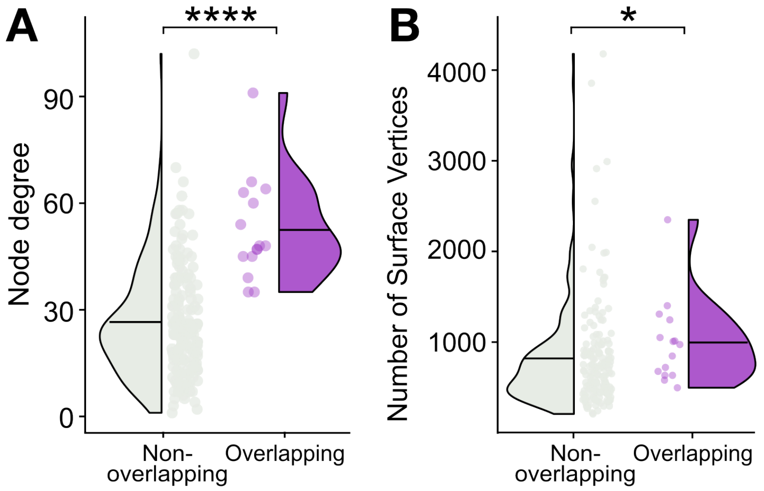

Figure S8: Overlapping regions identified by `OSLOM_30` have a greater degree and are larger than non-overlapping regions on average. A. The distributions of nodal degree are shown for overlapping regions (purple, 15 regions) and non-overlapping regions (grey, 164 regions) as raincloud plots. ****, $P = 3 \times 10^{-7}$, unpaired Wilcox rank-sum test between overlapping vs. non-overlapping degree (i.e., sum of log-transformed streamline counts per region). **B.** The distributions of cortical surface vertices contained within each overlapping region (purple, 15 regions) or non-overlapping region (grey, 164 regions) are shown as raincloud plots. *, $P = 0.03$, unpaired Wilcox rank-sum test between overlapping vs. non-overlapping vertex counts.

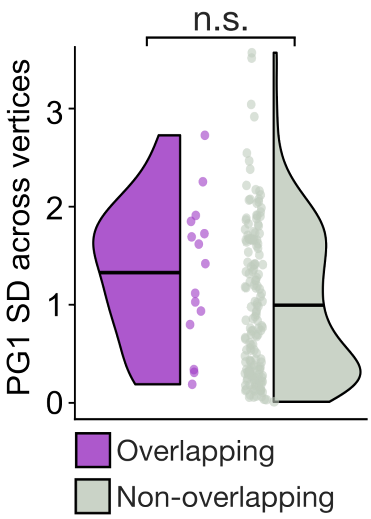

Figure S9: There is no difference in the vertex-wise SD of first principal gradient (PG1) values between overlapping vs. non-overlapping regions. The distributions of PG1 SD (across vertices) within each region are shown as raincloud plots for overlapping regions (purple, 15 regions) or non-overlapping regions (grey, 164 regions). n.s., $P > 0.05$, unpaired Wilcox rank-sum test between overlapping vs. non-overlapping PG1 SD values.

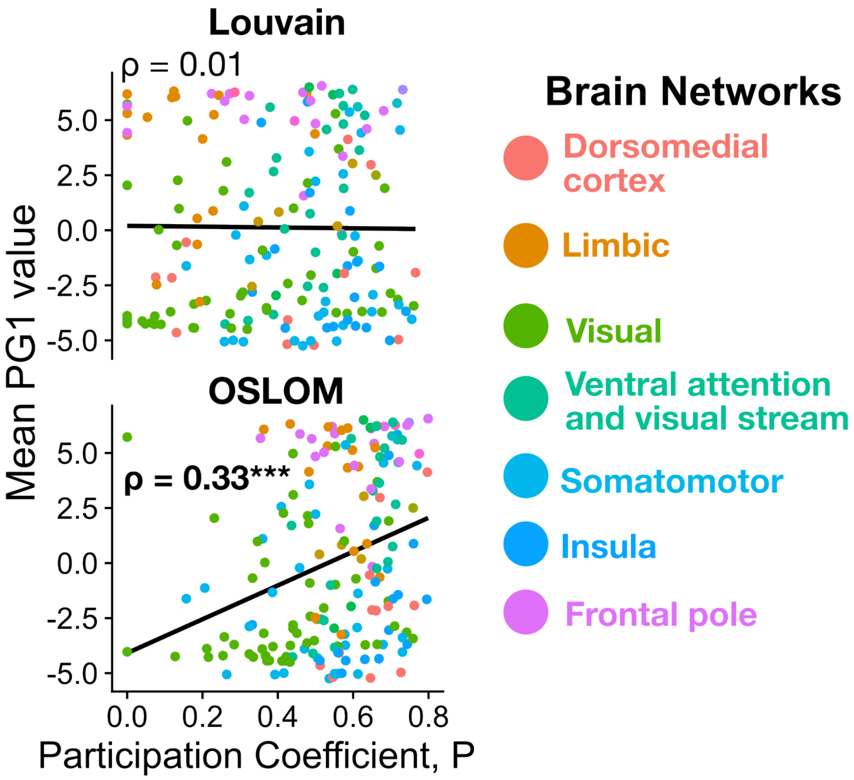

Figure S10: The participation coefficient from the OSLOM decomposition, but not the Louvain decomposition, is significantly correlated with the first principal gradient of functional connectivity (PG1) across the right hemisphere cortex. For each node in the 180-region HCP–MMP1 atlas, its mean PG1 value is compared with its participation coefficient from either Louvain (P_{Louvain}) or OSLOM (P_{OSLOM}) partitioning. Spearman's ρ is shown for each comparison; ***, $P < 0.001$.
